# Resf1 is a compound G4 quadruplex-associated tumor suppressor for triple negative breast cancer

**DOI:** 10.1371/journal.pgen.1011236

**Published:** 2024-05-09

**Authors:** Megan R. Majocha, Devin E. Jackson, Ngoc-Han Ha, Ruhul Amin, Marie Pangrácová, Christina R. Ross, Howard H. Yang, Maxwell P. Lee, Kent W. Hunter

**Affiliations:** 1 Laboratory of Cancer Biology and Genetics, National Cancer Institute, National Institutes of Health, Bethesda, Maryland, United States of America; 2 Lombardi Comprehensive Cancer Center, Georgetown University, Washington DC, United States of America; The Kansas University Medical Center, UNITED STATES

## Abstract

Patients with ER-negative breast cancer have the worst prognosis of all breast cancer subtypes, often experiencing rapid recurrence or progression to metastatic disease shortly after diagnosis. Given that metastasis is the primary cause of mortality in most solid tumors, understanding metastatic biology is crucial for effective intervention. Using a mouse systems genetics approach, we previously identified 12 genes associated with metastatic susceptibility. Here, we extend those studies to identify *Resf1*, a poorly characterized gene, as a novel metastasis susceptibility gene in ER- breast cancer. *Resf1* is a large, unstructured protein with an evolutionarily conserved intron-exon structure, but with poor amino acid conservation. CRISPR or gene trap mouse models crossed to the Polyoma Middle-T antigen genetically engineered mouse model (MMTV-PyMT) demonstrated that reduction of *Resf1* resulted in a significant increase in tumor growth, a shortened overall survival time, and increased incidence and number of lung metastases, consistent with patient data. Furthermore, an analysis of matched tail and primary tissues revealed loss of the wildtype copy in tumor tissue, consistent with *Resf1* being a tumor suppressor. Mechanistic analysis revealed a potential role of *Resf1* in transcriptional control through association with compound G4 quadruplexes in expressed sequences, particularly those associated with ribosomal biogenesis. These results suggest that loss of *Resf1* enhances tumor progression in ER- breast cancer through multiple alterations in both transcriptional and translational control.

## Author summary

Metastasis is the major cause of breast cancer deaths. However, unlike the primary tumor, acquired mutations do not appear to play a significant role in the development of metastatic disease. A person’s individual ancestry, however, does significantly influence metastatic efficiency. Our laboratory therefore seeks to identify and characterize the differences in inherited genes that increase or decrease metastatic burden. One inherited gene that changes metastatic efficiency is the poorly characterized gene Resf1. This gene encodes a large protein that has very little predicted structure. Reduction or loss of Resf1 accelerates tumor growth and increases the incidence and extent of metastatic tumors in the lung. Analysis here suggests that Resf1 protein is associated with DNA sequences that can form a DNA “knot”-like structure known as a G4 quadruplex. The association of Resf1 with these structures may be influencing the ability of tumor cells to undergo the epithelial-to-mesenchymal transition, which is thought to be critical for metastatic progression. Reduction or loss of Resf1 may therefore be accelerating the terminal steps of breast cancer progression.

## Introduction

Breast cancer is the most commonly diagnosed cancer and the second-leading cause of cancer-related death among women in the United States [1]. As the primary tumor is typically surgically removed upon detection, most of these deaths result from the effects of metastatic disease. This is best highlighted by the fact that the current five-year survival rate of patients with localized disease is approximately 90%, but drops to 31% when patients develop distant metastatic disease [1]. Thus, there is a critical need for therapies that can effectively prevent metastasis and/or treat established metastatic tumors. To improve patient outcomes, it is essential to identify bottlenecks and vulnerabilities within the metastatic cascade and in established secondary tumors. Addressing these factors is crucial for preventing or eliminating the lesions that are the proximal cause of most breast cancer-associated morbidity and mortality.

Despite considerable advances in our understanding of the cellular biology of metastases, the molecular mechanisms underlying this process remain poorly understood. The current leading theory is the Nowell Hypothesis [2], which posits that metastatic capacity is achieved through the cumulative acquisition of metastatic traits due to the somatic evolution of tumor cells over time. However, this hypothesis does not entirely align with all experimental data. Recent sequencing studies of metastatic cancers, derived from both mouse and human matched primary and metastatic samples, have failed to identify high-frequency metastasis driver genes [3]. These findings suggest that metastatic disease may be driven more by metastasis-associated transcriptional programs mediated by changes in gene copy number, structural rearrangements, and responses to specific environmental conditions, rather than constitutional activation or inactivation of specific pathways. Unfortunately, the absence of point mutations in metastasis driver genes complicates their identification, as they lack a somatic “flag post” by which to detect them.

An alternative approach to identifying metastasis modifiers uses population genetics to pinpoint metastasis susceptibility genes. Most inherited polymorphisms within species are located in non-coding sequences [4]. Variations in most population phenotypes, including disease (excluding high-penetrant mutation carriers like Li-Fraumeni Syndrome), are believed to arise from transcriptomic changes caused by polymorphisms within gene regulatory elements [5]. Thus, patients sharing identical oncogenic driver mutations and conventional clinical parameters may exhibit varying metastatic predispositions due to differences in their inherited transcriptional states. The identification of polymorphic metastasis susceptibility genes that can alter regulatory networks and transcriptional states through classical meiotic genetic screens, coupled with newer genomic technologies, offers an alternative method for gene identification. This approach utilizes polymorphic, rather than somatically altered, sequences as the genomic “flag post.”

To capture the effects of human diversity and the underlying causes of metastatic breast cancer, our laboratory employs a mouse systems genetics approach. The FVB/NJ-TgN(MMTV-PyMT)^634Mul^ (hereafter, MMTV-PyMT) mouse model is a highly metastatic and frequently used model of human breast cancer. This model most closely resembles the luminal subtype and displays characteristics similar to human breast cancer, including the gradual loss of hormone receptors. MMTV-PyMT animals exhibit a 100% mammary tumor penetrance at 9 weeks of age, with 85% developing pulmonary metastases within 100 days [6]. By breeding this model to a variety of inbred strains of mice to mimic human population diversity, we previously demonstrated that genetic backgrounds significantly influence the ability to progress to metastatic disease [7]. Subsequent mapping crosses, coupled with various “-omic” technologies, revealed polymorphic candidate genes associated with metastatic progression. In one cross involving the Japanese-derived MOLF/EiJ mouse strain, five genes on the distal end of mouse chromosome 6 were implicated in the progression of ER-negative (ER-) breast cancer, including the circadian rhythm transcription factor *Arntl2* [8]. In this study, we expand upon these previous findings and provide evidence that an additional gene within this interval, Retroviral silencing factor 1 (*Resf1*), is a potential tumor suppressor and metastasis modifier in ER-negative breast cancer.

## Results

### Identification of *Resf1* as a potential metastasis susceptibility gene

**Fig 1:**
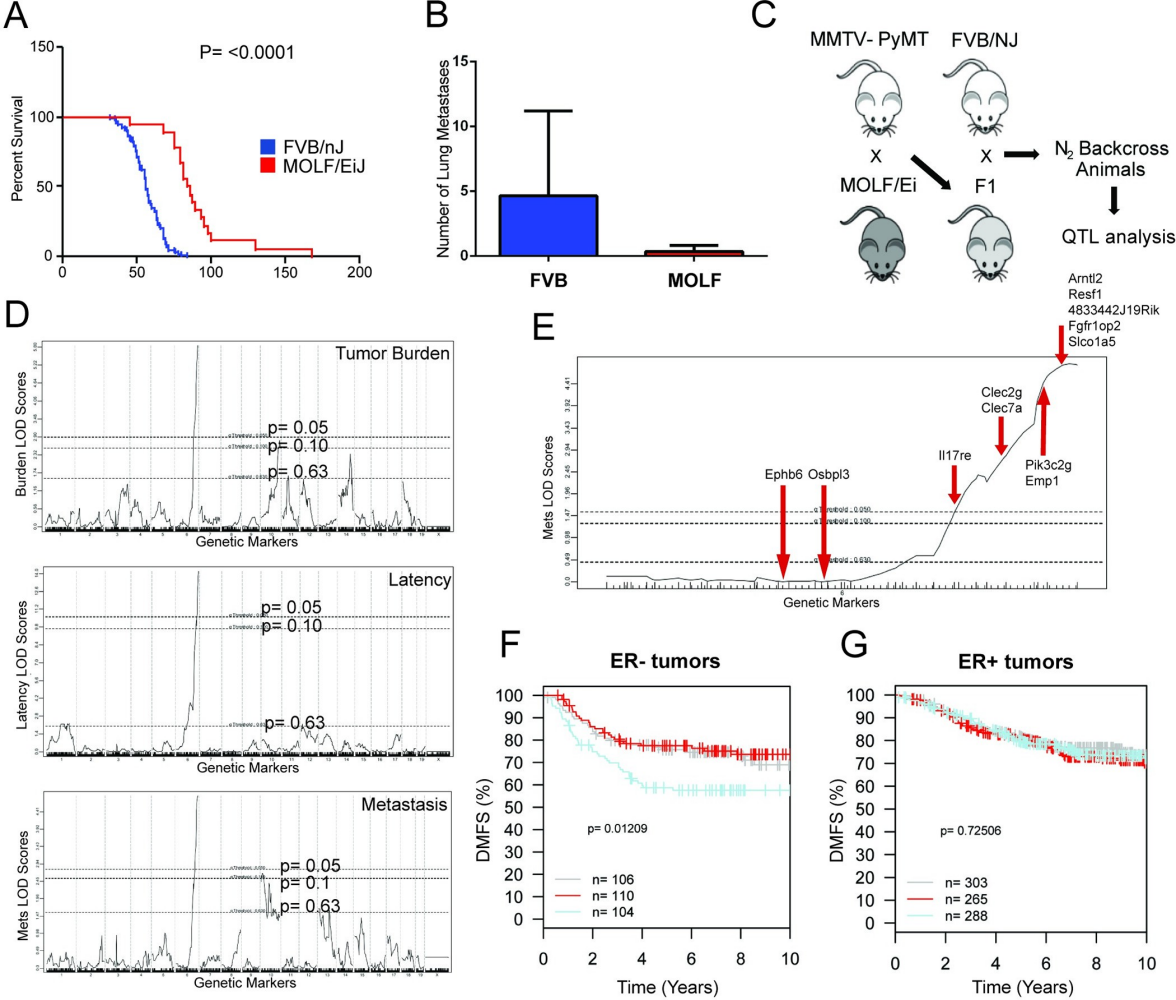
Mouse strains with differing metastatic capacity identify potential metastasis susceptibility genes on chromosome 6. Fig 1 is adapted from Ha et al.[8]. (A) Kaplan-Meier survival plot of FVB/NJ x (MOLF/EiJ x MMTV-PyMT) mice. The p-value is based on a log rank test. Comparisons are of both MOLF/EiJ and FVB/NJ F1 mice after crossing with MMTV/PyMT. (B) Pulmonary surface metastases were counted after MMTV-PyMT cross with each strain. P-value is calculated by Mann-Whitney test. (C) A schematic of our breeding scheme to segregate chromosomes during meiosis. (D) Genetic association analysis based LOD scores (y-axis) observed for metastasis, tumor latency, and tumor burden, with the x-axis representing chromosomes lined up from head to tail. The upper horizontal dashed line represents a p-value significance threshold of 0.05 after permutation testing. (E) Zoomed-in region of distal chromosome 6 containing candidate genes for study. (F) GOBO analysis of the 12 gene signature identified from gene expression-tumor phenotype correlation analysis. DMFS (distant metastasis-free survival) plotted as a Kaplan-Meier survival curve shows a significant reduction in survival in ER+ breast cancer and (G) ER- breast cancer with lower expression. High (blue), intermediate (red), and low (gray) levels of gene expression-tumor phenotype correlation gene signature.

Mouse outcrosses with various strains of inbred and wild-derived strains demonstrated that the inherited genome significantly affected tumor and metastatic burden outcomes [7]. One of the most significantly different tumorigenic burdens among the inbred mice used in the outcross was MOLF/EiJ, a Japanese wild-derived strain (Fig 1A). Compared with FVB/NJ control mice, MOLF/EiJ mice developed tumors with significantly extended latency and exhibited reduced tumor growth and pulmonary metastases (Fig 1B) [8] [9]. To investigate the genetic basis of this observation, a MOLF/EiJ x FVB/MMTV-PyMT cross was performed followed by a backcross to FVB/NJ to segregate the genome during meiosis, resulting in N = 171 backcross (N_2_) animals (Fig 1C). To identify regions of the MOLF/EiJ genome associated with changes in tumor phenotypes, these N_2_ animals were genotyped using the Illumina Mouse Medium Density Linkage Panel. Logarithm of odds (LOD) scores displayed a significant correlation in all 3 tumor phenotypes (tumor burden, tumor latency, and metastatic burden) at the distal portion of chromosome 6 (Fig 1D). Thus, there may be metastasis modifier genes at distal chromosome 6 that affect all 3 tumor phenotypes based on the genetic association between the traits mapped to the locus.

To identify candidate genes in this region of chromosome 6, gene expression-tumor phenotype correlation analysis was performed on 134 tumors from N_2_ animals, as previously described [8]. Briefly, each of the 134 tumors was screened for genes at the peak of the distal chromosome 6 peak that was associated with distant metastasis-free survival (DMFS) in this population using Affymetrix ST 1.0 array analysis [8]. Using a significance threshold of p = 0.05, 10 genes associated with metastatic outcome were identified (Fig 1E).

To further validate the potential role of these 10 genes in human breast cancer, the candidate genes were used to generate a gene signature that could be applied to human breast cancer gene expression data. The human orthologs for each gene were identified and a human gene signature was generated by assigning weights and directionality based on the hazard ratios determined in the mouse Affymetrix data [8]. The GOBO (Gene Expression-Based Outcome for Breast Cancer Online) web tool [10] was used to stratify the disease outcome of these 10 genes in human breast cancer as well as determine their associations with DMFS. A significant association with DMFS was observed in ER- breast cancer (Fig 1F), but significance was not observed in ER-positive (ER+) breast cancer (Fig 1G). This is consistent with one or more previously studied genes within this signature playing a significant role in the progression of this highly malignant subtype of human breast cancer.

Examination of the location of the 10 candidate genes on the chromosome revealed that 5 genes (*Arntl2*, *2810474O19Rik/Resf1*, *4833442J19Rik/Etfbkmt*, *Fgfr1op2*, *Slco1a5*) were located at the peak of the LOD score for all 3 tumor phenotypes [8]. Examination of the Kaplan-Meier (KM) plots for DMFS in the mouse data revealed the greatest stratification for survival for *Arntl2*, *Resf1*, and *Etfbkmt* (S1 Fig), which were selected for further analysis. The validation and characterization of the role of *Arntl2* in breast cancer metastasis was previously described by our group [8]. The potential role of *Resf1* in breast cancer metastasis is discussed here.

#### Polymorphisms in the *Resf1* promoter alter gene expression

**Fig 2:**
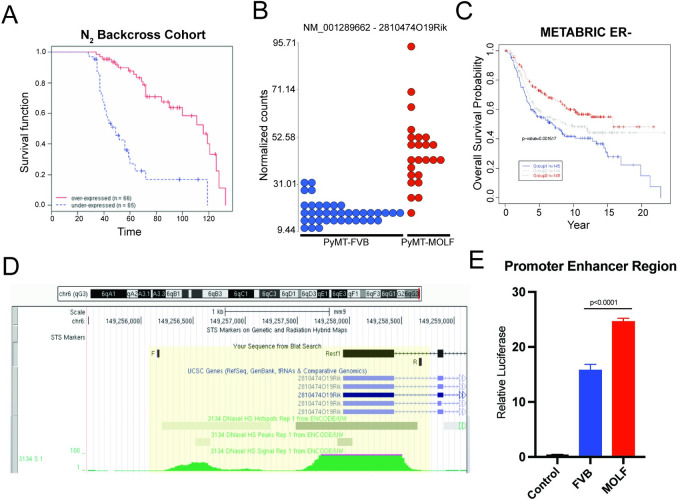
*RESF1* expression levels corroborate human data and are associated with upstream SNPs. (A) From the N_2_ backcross cohort in Fig 1C, 131 mice were categorized into *Resf1* over and under-expressed groups and plotted as Kaplan-Meier survival curves showing worse DMFS survival in animals under-expressing *Resf1*. (B) RNA-seq of paired spontaneous mammary tumors and lung metastases from FVB/NJ and MOLF/EiJ mice that were crossed with MMTV-PyMT were analyzed for *Resf1* expression levels and shows higher expression levels in the less metastatic MOLF/EiJ strain. (C) Query of the METABRIC human breast cancer tumor database shows significant reduction in survival at lower expression levels of *RESF1*. High (red), intermediate (gray), low (blue). (D) Query of the UCSC BLAT Genome Browser for the 5’ UTR and upstream region of *Resf1* displays DHS peaks (green) in the highlighted yellow area. Included are locations of primers used for PCR and cloning of the promoter enhancer region. (E) Upstream regions of *Resf1* in FVB/NJ and MOLF/EiJ were cloned into the luciferase pGL4.23 reporter plasmid, and dually transfected with the Renilla hRluc plasmid into HEK293T cells, with empty vector as a negative control. Values show ratio of Firefly luciferase to Renilla in empty vector control, FVB/NJ upstream region, and MOLF/EiJ upstream region. P-value based on unpaired t-test.

The gene expression-tumor phenotype correlation analysis indicated that variation in *Resf1* expression might contribute to metastatic progression, consistent with the hypothesis that most inherited susceptibility is mediated by changes in gene expression rather than coding mutations [11,12]. Indeed, KM curves of mouse tumor expression data demonstrated that animals with lower *Resf1* had worse survival (Fig 2A). Furthermore, RNA-sequencing (RNA-seq) analysis comparing FVB/NJ PyMT tumors versus MOLF/EiJ x FVB/NJ F1 tumors showed that the more highly metastatic homozygous FVB/NJ tumors had lower *Resf1* expression (Fig 2B). The RNA expression data for human breast cancer available through the METABRIC consortium were consistent with this observation, as patients with ER- tumors and lower expression of *RESF1* had worse outcomes than those with intermediate or high levels (Fig 2C). Furthermore, published DNase hypersensitivity (DHS) mapping data from the 3134 mouse mammary tumor cell line was mapped onto the *Resf1* sequence and revealed two DHS sites, proximal to and overlapping the 5’ UTR, suggesting a potential promoter function in this region (Fig 2D). Examination of the sequence across this region revealed the presence of 28 single nucleotide polymorphisms (SNPs) between FVB/NJ and MOLF/EiJ within the DHS sites upstream of and overlapping the 5’ UTR (S2A Fig). Promoter reporter assays of the 5’ UTR containing both DHS sites revealed that the MOLF/EiJ allele had significantly higher transcriptional activity, confirming a functional consequence of the inherited promoter variants (Figs 2E and S2B). These results suggest that inherited variants in the transcriptional control elements of the *Resf1* gene may drive differential expression from the FVB/NJ and MOLF/EiJ alleles that alter metastatic capacity.

### Decreased *Resf1* expression is associated with increased autochthonous metastasis

**Fig 3:**
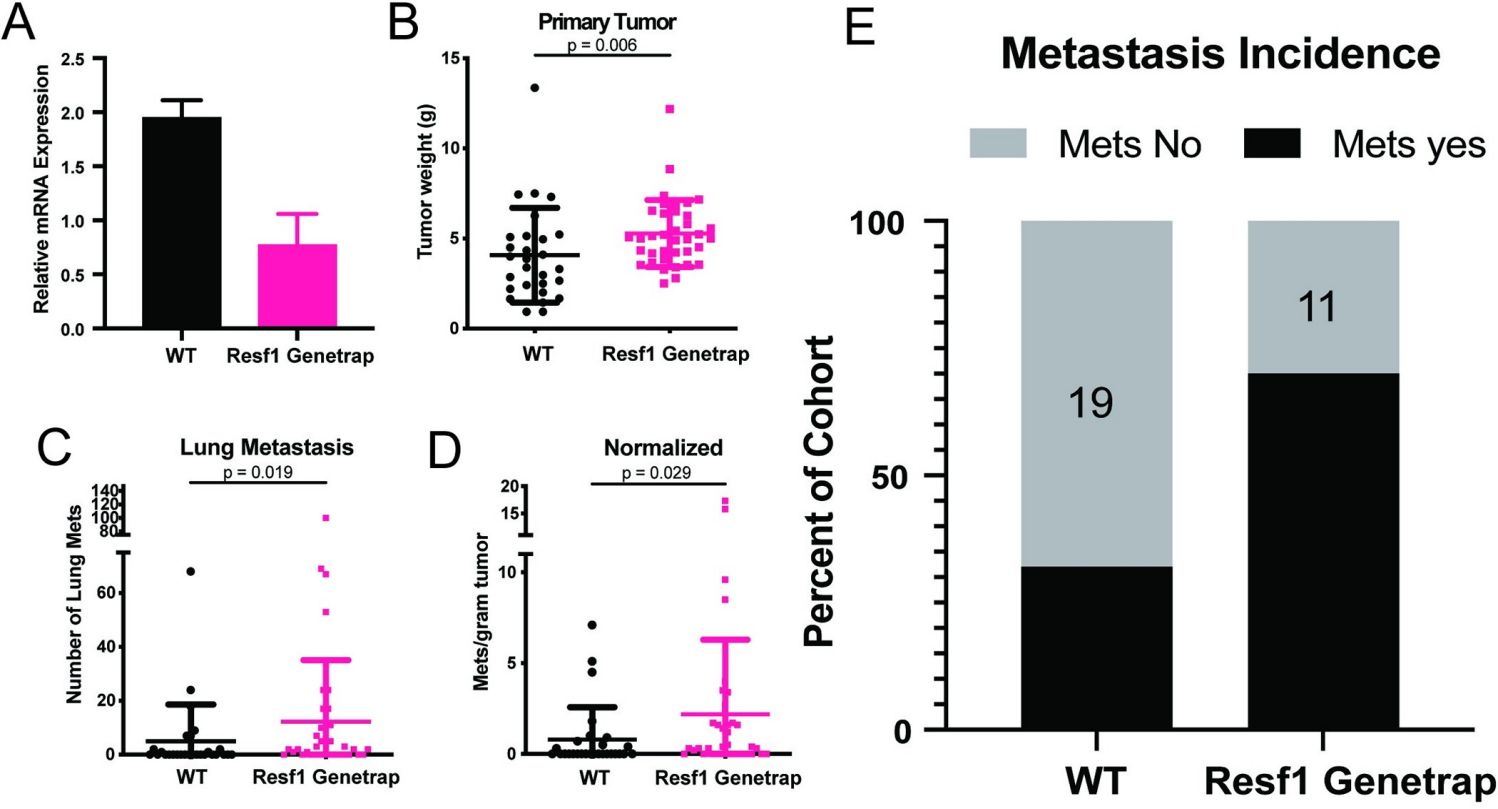
Lower stromal levels of *Resf1* increase metastatic burden and incidence. (A) qRT-PCR analysis of *Resf1* in primary tumors from WT and genetrap hypomorph crossed with MMTV/PyMT mice (n = 5 per group). Control and hypomorph mice were crossed with MMTV-PyMT mice to induce spontaneous mammary tumors and pulmonary metastases (B-E). (B) Primary mammary fatpad tumors were collected and weighed for WT (n = 27) and hypomorph (n = 38) and resulted in significantly larger tumors in hypomorph mice, p-value calculated by Mann-Whitney test. (C) Surface lung metastases were counted, resulting in more metastases in hypomorph mice, p-value calculated by Mann-Whitney test. (D) Normalization of lung metastases per gram tumor to account for larger tumor size remained significantly higher in hypomorph mice, p-value calculated by Mann-Whitney test. (E) Metastatic incidence was higher in hypomorph mice compared to control.

To validate *Resf1* as a metastasis modifier and to determine its disease phenotype *in vivo*, a *Resf1* genetically engineered mouse model (GEMM) generated by an enhancer/gene trap insertion was used. The C57BL/6-Et(EGFP/Cre)^16255Rdav^ mouse model (hereafter, the gene trap model) was obtained from the Mutant Mouse Resource & Research Centers. This model was generated by lentiviral gene trap construct integration into the first exon of *Resf1*, resulting in the expression of an EGFP-cre fusion and the truncation of the *Resf1* message (www.mmrrc.org/catalog/sds.php?mmrrc_id=34574). qRT-PCR analysis of mammary tumor tissue from wildtype and gene trap x FVB/MMTV-PyMT littermates confirmed a reduction of *Resf1* mRNA (Fig 3A). Tumor weight and lung metastases of the gene trap x PyMT F1 mice were collected and assessed at the humane endpoint. The *Resf1* gene trap mice developed significantly larger primary tumors and significantly more lung metastases than wildtype littermate control mice, even when normalizing metastasis counts to account for the larger tumors (Fig 3B–3D). Furthermore, the incidence of metastases in the gene trap mice was significantly higher than in the control mice (67% vs. 36%, p = 0.05, Fisher’s exact test; Fig 3E), consistent with the poor survival observed in human patients with lower *RESF1* expression.

### Decreased *Resf1* expression in cell lines paradoxically reduces the metastatic capacity

The role of RESF1 in metastatic disease was further explored by using orthotopic transplants for spontaneous metastasis assays. We attempted to generate cell lines with stable *Resf1* overexpression but were unsuccessful, suggesting that there is a threshold beyond which *Resf1* expression negatively impacts cell viability. Therefore, subsequent efforts focused on shRNA-mediated knockdown (KD) of *Resf1*.

**Fig 4:**
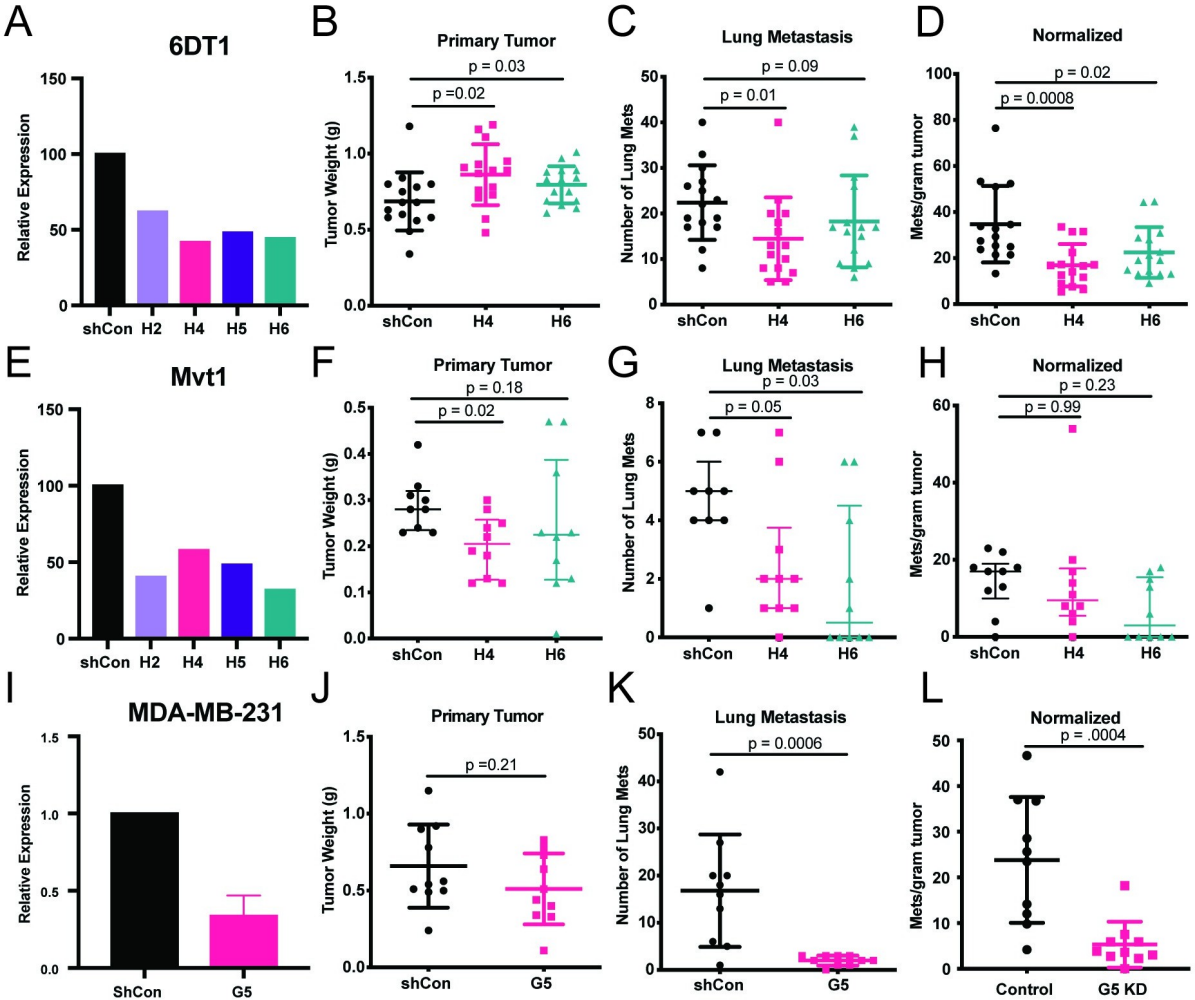
Decreased *Resf1* in cell lines paradoxically decreases metastatic capacity. (A) qRT-PCR analysis of 6DT1 shRNA-mediated *Resf1* stable KD cells. (B) Weight of primary tumors from 6DT1 Control (scramble), H4, and H6 cells orthotopically injected into the 4^th^ mammary fatpad of syngeneic FVB/NJ mice, n = 15 mice per group. (C) Surface pulmonary metastasis in mice from (B). (D) Pulmonary metastases normalized per gram tumor from (B). (E) qRT-PCR analysis of Mvt1 shRNA-mediated *Resf1* stable KD cells. (F) Weight of primary tumors from Mvt1 Control (scramble), H4, and H6 cells orthotopically injected into the 4^th^ mammary fatpad of syngeneic FVB/NJ mice, n = 10 mice per group. (G) Surface pulmonary metastasis in mice from (F). (H) Pulmonary metastases normalized per gram tumor from (F). (I) qRT-PCR analysis of MDA-MB-231 shRNA-mediated RESF1 stable KD cells. (J) Weight of primary tumors from MDA-MB-231 Control (scramble) and G5 KD cells orthotopically injected into the 4th mammary fatpad of nu/nu mice, n = 10 mice per group. (K) Surface pulmonary metastasis in mice from (J). (L) Pulmonary metastases normalized per gram tumor from (J). P-values in B-D and F-H calculated by ordinary one-way ANOVA with Dunnett’s multiple comparison test. P-values in J-L calculated by Mann-Whitney test.

*Resf1* was knocked down via stable shRNA transduction of 4 constructs into 6DT1 and Mvt1, both mouse cell lines syngeneic to FVB/NJ [13] (Fig 4A and 4E). *Resf1* KD was confirmed by qRT-PCR (Fig 4A and 4E). The H4 and H6 shRNAs displayed consistent KD in both cell lines and were used for further *in vivo* experiments. shScramble control and *Resf1* shRNA H4 and H6 cells were injected orthotopically into the 4th mammary fat pad of syngeneic FVB/NJ mice. Primary mammary tumors and lung metastases were collected and assessed after 28 days. Inconsistent primary tumor growth changes upon shRNA-mediated KD were observed for the 6DT1 and Mvt1 cell lines, indicating cell line-specific effects on tumor cell growth (Fig 4B and 4F). Surprisingly, both KD cell lines showed a reduction in lung metastases compared to the control (Fig 4C and 4G), in contrast to our expectations based on the human data and mouse gene trap x PyMT model, which formed more lung metastases (Fig 3B–3D).

Although previous studies have demonstrated that the MOLF/EiJ x PyMT backcross population approximates the diversity of human breast cancer subtypes at the transcriptional level [8] [14,15], the MMTV-Myc tumors from which the Mvt1 and 6DT1 cell lines derived are thought to most closely resemble the human luminal subclasses [16]. To determine whether the opposite metastatic capacity observed in these cell lines was due to either a species-specific effect or a subtype-specific effect, *RESF1* was stably knocked down (Fig 4I) in the highly malignant triple-negative MDA-MB-231 human cell line and orthotopically implanted into immunocompromised NU/J mice. KD of *RESF1* did not significantly alter primary tumor growth and significantly reduced lung metastasis, which is consistent with the findings from our 6DT1 and Mvt1 allografts (Fig 4J–4K). The difference between the cell line data and human patient or endogenous mouse data may therefore be the result of an artifact introduced into cells during the establishment of *ex vivo* cell lines. Alternatively, *Resf1* may have opposing functions at the primary and secondary site, similar to *Tgfβ* which has previously been shown to have tumor suppressive functions at the primary site but pro-tumorigeneic effects for tumor invasion and metatasis [17].

To further address this question and to control for potential off-target effects in the gene trap mouse model, a second *Resf1* GEMM from the KOMP project (C57BL/6NCrl-2810474O19Rik^em1(IMPC)Mbp/Mmucd^; hereafter referred to as the *Resf1* knockout (KO) mouse) was incorporated into the study. This GEMM carries a CRISPR-Cas9 mediated deletion of exon 4, which contains almost the entirety of the *Resf1* coding sequence, and results in a 50% reduction of *Resf1* mRNA levels. The *Resf1* KO mouse was bred to the PyMT model to compare the tumor phenotypes of PyMT^+^/Resf1^wt/wt^ and PyMT^+^/Resf1^wt/KO^ compound heterozygous animals. Consistent with the gene trap model, the reduction of *Resf1* by CRISPR-mediated deletion was associated with increased tumor burden, more rapid tumor growth, and an increase in the number of pulmonary metastases (S3A–S3D Fig).

To test for a potential role for *Resf1* in the tumor stroma, orthotopic transplants of 6DT1 cells into the mammary fat pads of (FVB/NJ x *Resf1* KO) F_1_ or wildtype FVB littermate animals were performed. However, no differences were observed between the two genotypes, suggesting *Resf1* functions in a tumor-autonomous fashion (S3E–S3H Fig). Moreover, genotyping analysis of matched tail and primary tumor samples from PyMT^+^/Resf1^wt/wt^ and PyMT^+^/Resf1^wt/KO^ F_1_ mice showed the loss of the wildtype allele in many of the animals (S3I Fig), supporting the role of *Resf1* as a tumor suppressor. This is consistent with previous studies that suggest that *Resf1* is a rare tumor suppressor gene [18]. In contrast, RNA-seq analysis of matched FVB-PyMT tumors and metastases demonstrated higher *Resf1* expression in metastases, which suggests a potential pro-metastatic role at the secondary site (S3J Fig). Combined, these data suggest a complex dual role of *Resf1* in tumor progression analogous to *TGFβ*, with functions as a tumor suppressor in the primary tumor but metastatic promoting abilities in the secondary site.

### RESF1 is a nucleoplasmic protein

The Human Protein Atlas revealed RESF1’s primary localization in nucleoli, aligning with its potential role in ribosomal biogenesis. Our immunofluorescence analysis using the same antibodies confirmed this localization (S4A and S4B Fig). However, when epitope-tagged mouse or human RESF1 was transiently transfected, *Resf1* was excluded from nucleoli and instead localized to the nucleoplasm (S4C Fig), calling into question the specificity of the antibodies used for immunofluorescence staining. To address this, we generated rabbit monoclonal antibodies against the mouse RESF1 ortholog, validated by western blot in transiently transfected cells and *Resf1* CRISPR-KO mouse cell lines (S4D–S4E Fig). In contrast to human antibodies (15622 and 13816), the recombinant anti-mouse antibodies (15A4 and 186C7) stained nuclear speckles (S4F Fig). However, none of these antibodies, including the human 13816 antibody, exhibited signal loss CRISPR-KO cell lines, suggesting off-target reactivity in immunofluorescence conditions.

To resolve the nuclear localization of RESF1, subcellular fractionation was performed. Fractionation of 4T1 and 6DT1 mouse cancer cells revealed that endogenous RESF1 was predominantly located in the nuclear fraction (S4G Fig). Next, we used CRISPR to add dTAG-2xHA to the C-terminus of endogenous RESF1. Immunofluorescence analysis of the HA tag showed RESF1-dTAG-2xHA staining in the nucleoplasm (S4H Fig). Nucleolar and nuclear speckle staining was also observed in a subset of cells, suggesting that RESF1 can occupy these structures. This also raises the possibility that the anti-RESF1 antibodies may be recognizing a RESF1-associated structure in addition to the RESF1 protein itself, though further work would be required to address this possibility.

### RESF1 is associated with compound G4 quadruplexes

**Fig 5:**
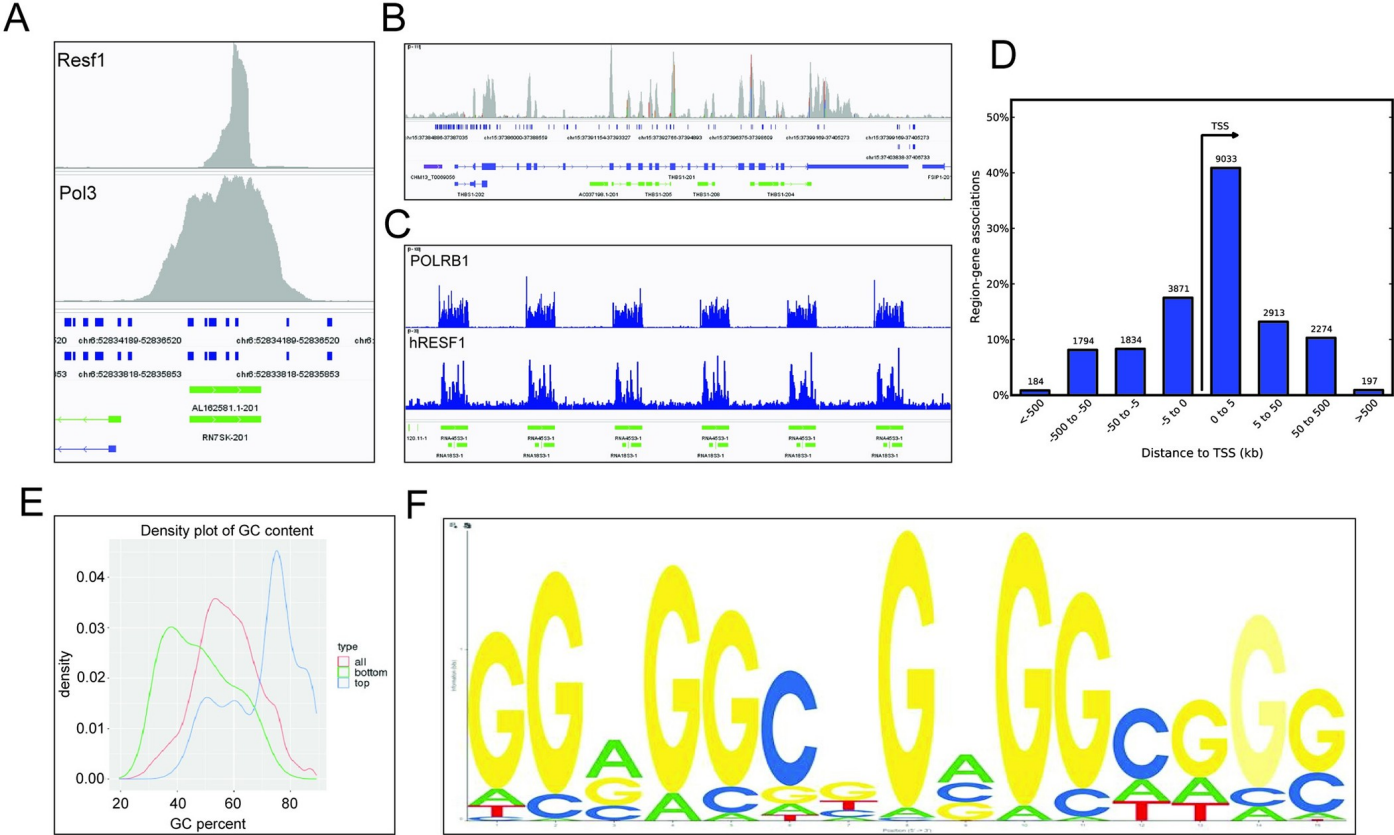
RESF1 is associated with many regions across the genome. BioTAP XL data was mapped against the T2Tv2 human genome and showed significant associations between Resf1 and (A) 7S RNAs, (B) exons of protein-coding genes, and (C) ribosomal RNA repeats. (D) Histogram graph showing the majority of the BioTAP XL sites within 0–5 kb downstream of the TSS. (E) Comparisons of the top 1000 RESF1 BioTAP XL binding sites versus the bottom 1000 sites revealed a significant association between RESF1 and GC-enriched sequences. (F) Identification of the de novo motif with the potential to form G4s within the BioTAP XL sites.

To further dissect the role of RESF1 in the nucleus, chromatin association analysis was performed. Fortuitously, a search of the Sequence Read Archive (SRA) public database revealed a proximity labeling BioTAP XL [19] study of the human ortholog of RESF1 deposited by the Elledge laboratory (PRJNA509912). This experiment consisted of a tetracycline-inducible RESF1 construct fused to an endogenous biotinylation targeting signal to biotinylate and isolate RESF1 chromatin. Mapping this data against the T2Tv2 human genome revealed 15882 sites significantly associated with RESF1 after an FDR cut-off of 0.05. Examination of the sites revealed significant associations of RESF1 across highly expressed 7S RNAs (Fig 5A), exons of protein-coding genes (Fig 5B), and ribosomal RNA repeats (Fig 5C). The BioTAP XL data was then remapped on hg38 and analyzed with the GREAT tool [20]. 14,929 BioTAP XL sites were mapped to the hg38 genome and were associated with 61% (11,546/18,777) of the annotated genes in this genome build. The majority of the BioTAP XL sites were within 0–5 kb downstream of the transcription start site (TSS), suggesting a potential association with transcribed sequences (Fig 5D). Gene Ontology analysis revealed a significant association of RESF1 with genes involved with translation, noncoding RNA, and ribosomal biogenesis (S5A and S5B Fig).

Further examination of some of the most significantly associated RESF1 sites revealed highly GC-enriched sequences. Comparisons of the top 1000 versus the bottom 1000 demonstrated that the most significant RESF1-associated sites had approximately 70% GC content and 70.3% (11179/15822) overlapped CpG islands in the T2Tv2 genome (Fig 5E). De novo motif detection identified GGVGGCNGVGGHDGS (Fig 5F) as an enriched potential motif within the BioTAP XL sites. This motif resembled sequences that have the potential to form DNA G4 quadruplexes, a nucleic secondary structure formed by stacking two or more Hoogsteen base-paired guanine tetrads to form a DNA “knot”. DNA G4 quadruplexes have been implicated in a number of nuclear activities, including transcriptional control, DNA stability, and RNA splicing [21,22,23,24]. DNA G4 predictions were therefore calculated for the T2Tv2 transcriptome using the R package pqsfinder and compared to the BioTAP XL data. 93.4% of the BioTAP XL-associated sites (14842/15882) overlapped predicted DNA G4 quadruplexes in the transcriptome, supporting the possibility that RESF1 is primarily associated with quadruplexes in transcribed sequences rather than DNA G4 quadruplexes associated with transcriptional control elements.

**Fig 6:**
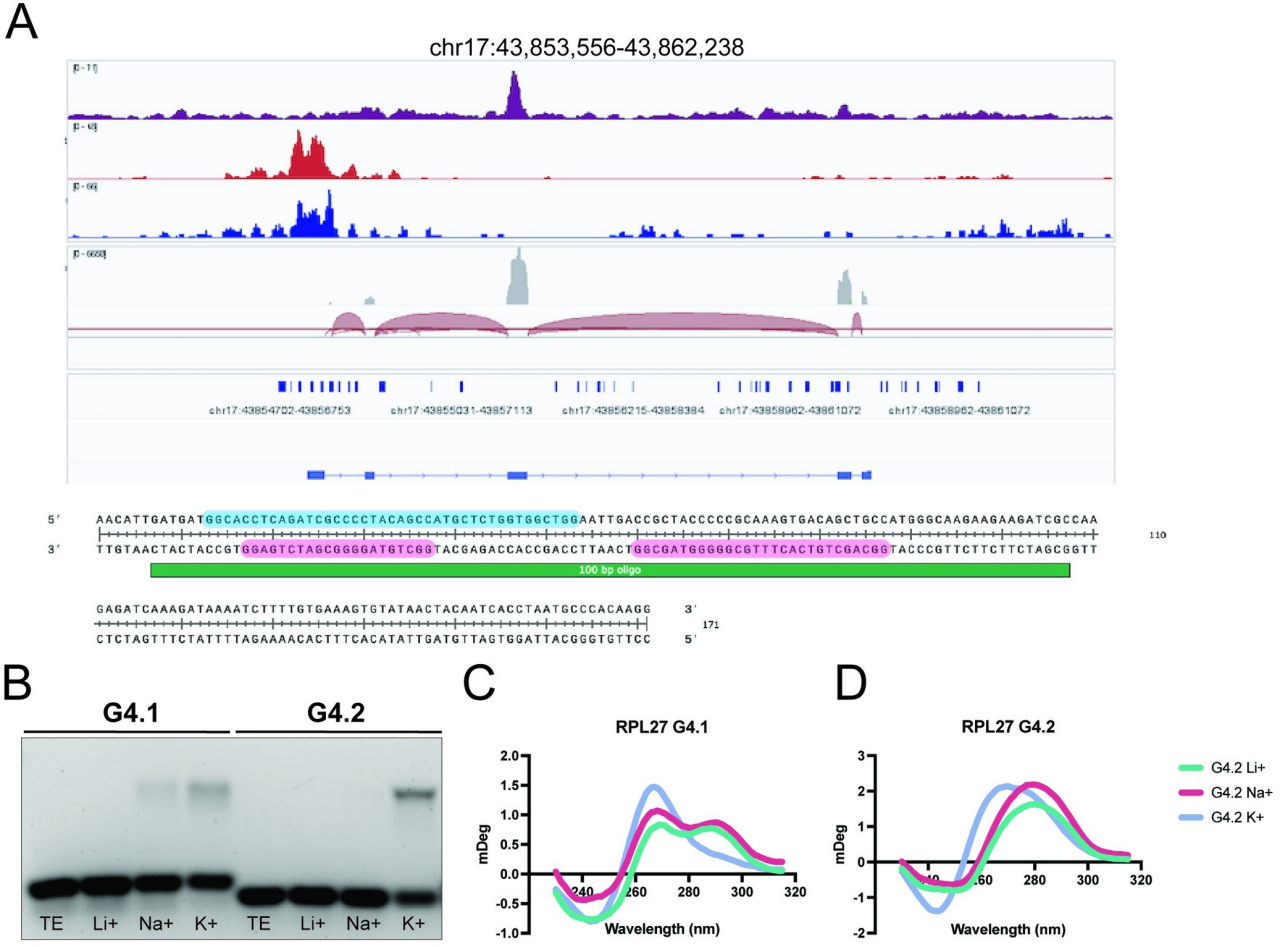
RESF1 is associated with G4 quadruplexes on the template and non-template strands. (A) BioTAP XL analysis showing a significant association between RESF1 and RPL27 exon 3 containing G4s on both strands. (B) Gel mobility shift assays with RPL27 compound G4s (G4.1 and G4.2) on the template strand. (C-D) Circular dichroism analysis of RPL27 compound G4s on the template strand with the presence of lithium, sodium, or potassium ions.

To better understand the association of RESF1 with transcribed sequences, the ribosomal *RPL27* gene was selected for further study. *RPL27* showed a strong RESF1-associated peak over exon 3 (Fig 6A). Moreover, the promoter and 5’ UTR of *RPL27* is associated with DNA G4 quadruplex as well as iMotif structures [25] but is not associated with RESF1, consistent with a non-promoter/enhancer role for RESF1 in transcription. Exon 3 of RPL27 was reanalyzed for potential G4 quadruplexes spanning up to 45 base pairs with the web tool QGRS Mapper. QGRS Mapper predicted tandem DNA G4 quadruplexes on the template strand of exon 3 and a single predicted quadruplex on the non-template strand (Fig 6A). Other *RPL27* exons contained only a single predicted DNA G4 quadruplex on the non-template strand. Electrophoretic gel shift assays of an oligo spanning this predicted DNA G4 were not consistent with the formation of a quadruplex *in vitro*. However, DNA secondary structure prediction indicated this sequence had a strong predilection to form a DNA hairpin, which was supported by circular dichroism analysis. Previous studies mapping G4 quadruplexes in cytoplasmic RNA (SRA accession number PRJNA673726) [26] did reveal the presence of a RNA G4 quadruplex in exon 3 of the RPL27 mRNA, suggesting the potential for quadruplex formation in more physiological conditions (S5C Fig). Circular dichroism and DNA G4 gel shift assays using oligos spanning each of the predicted exon 3 quadruplex sequences on the template strand demonstrated a shift in the presence of potassium ions, but not sodium or lithium, consistent with the formation of DNA G4 quadruplexes (Fig 6B–6D). Together, these data suggest that RESF1 may be associated with transcribed sequences containing one or more DNA G4 quadruplexes on both strands.

To further examine this possibility, DNA G4 quadruplex analysis was performed on the top 1000 RESF1 binding sites with QGRS Mapper. 96.6% (966/1000) of the RESF1-associated sites contained QGRS Mapper-predicted quadruplexes on both DNA strands, with 95.4% of sites containing 2 or more predicted quadruplexes on at least one strand. The DNA G4 quadruplexes predicted were highly enriched for quadruplexes containing only two guanine tetrads, which are thought to be less stable than quadruplexes with 3 or more tetrads. Combined, these data suggest that RESF1 is associated predominately with transcribed sequences containing one or more potential duplex DNA G4 quadruplex structures on both DNA strands.

### RESF1 metastatic phenotype is not associated with global alterations in translation

**Fig 7:**
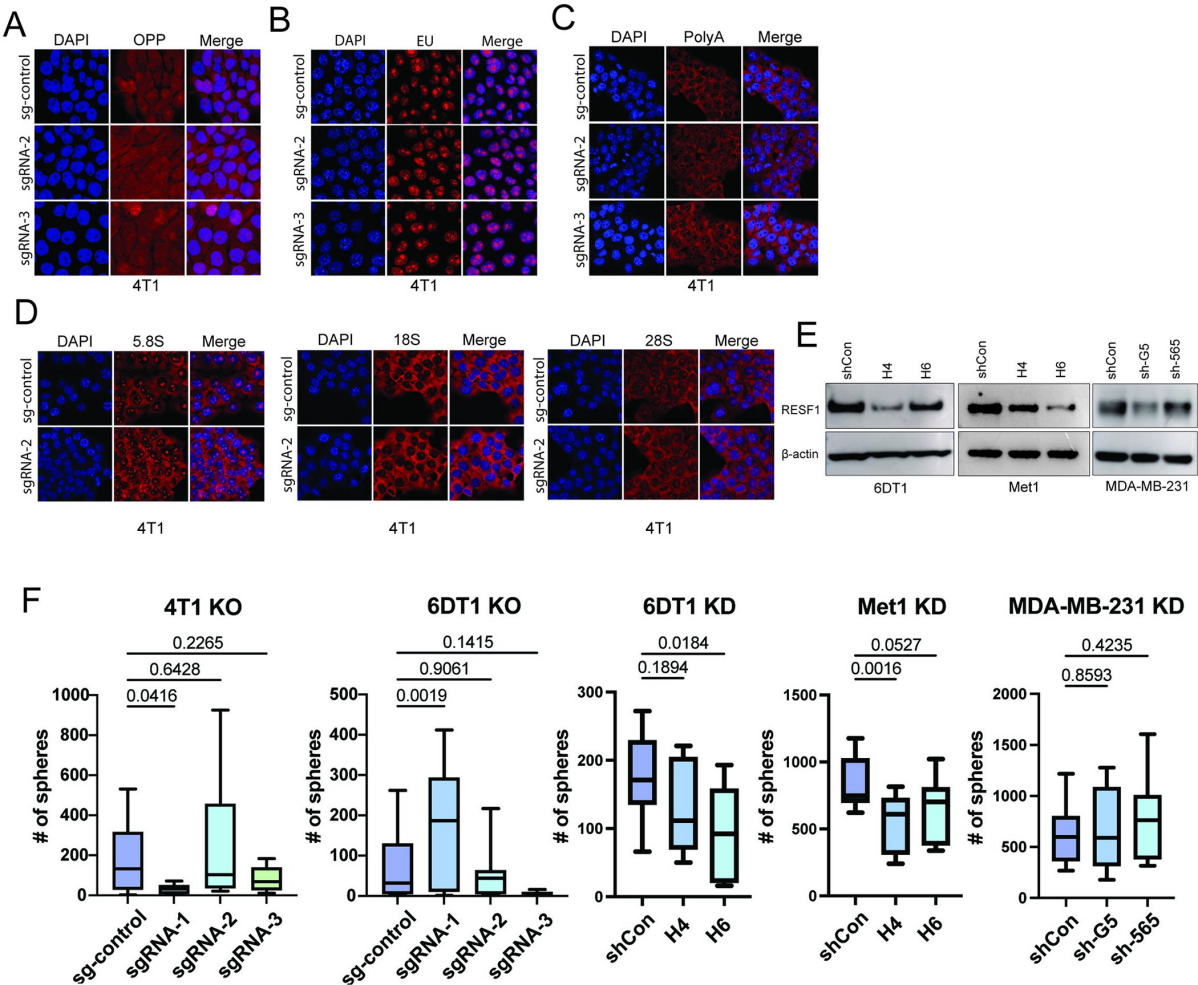
*Resf1* is not associated with its known functions in metastasis. Confocal fluorescence microscopy images of (A) a 30-minute OPP (o-propargyl-puromycin) pulse, (B) a 30-minute EU (ethinyl uridine) pulse, and (C) PolyA mRNA FISH, (D) 5.8S, 18S, and 28S rRNA FISH of 4T1 CRISPR KO cell lines. (E) Western blot analysis showing shRNA-mediated KDs of *Resf1* in 6DT1, Met1, and MDA-MB-231 cell lines. (F) Tumorsphere assays with 4T1 and 6DT1 CRISPR KO cells, 6DT1, Met1, and MDA-MB-231 KD cells. P-values were calculated by ordinary one-way ANOVA with Dunnett’s multiple comparison test.

RESF1 has been previously implicated in mRNA transport and protein production [27]. To determine if this activity accounted for the *Resf1* metastasis phenotype, *Resf1* CRISPR knockouts (KO) were generated in the 4T1 and 6DT1 cell lines (S4E Fig). O-propargyl-puromycin (OPP) assays were performed to examine potential global changes in protein synthesis. However, in contrast with published results, no difference in OPP signal between 4T1 or 6DT1 *Resf1* KO cells compared to the control was observed (Fig 7A). To assess nascent RNA production, an ethynyl uridine (EU) pulse assay to label actively transcribed RNAs was performed. In contrast to Ritter et al. [27], no increase in the labeling of cytoplasmic RNA was observed in *Resf1* KO or KD cells (Figs 7B and S6). PolyA FISH analysis also showed no significant changes between control and *Resf1* KO cell lines, suggesting the loss of RESF1 did not alter global mRNA production or transport (Fig 7C). Furthermore, mTOR antibody arrays to investigate alterations in translational efficiency (S7 Fig) and western blot analysis of critical canonical unfolded protein response (UPR) targets showed no significant changes between control and KD cell lines grown on glass or plastic culture surface with and without prior tunicamycin (TM) treatment for UPR activation (S8 Fig). Thus, RESF1 depletion does not appear to alter global protein translation in metastatic cells.

To determine whether ribosomal RNA is altered in *Resf1* KO cell lines, rRNA subunit RNA FISH was performed. We observed no significant differences in 5.8S, 18S, or 28S levels between *Resf1* control and KO cell lines (Fig 7D). Moreover, GSEA analysis of cell line RNA-seq data indicated significant suppression of ribosomal subunit transcripts in the KD cell lines (S9A–S9C Fig). Western blot analysis, however, revealed no significant difference in several ribosomal proteins except for a decrease in the RPL22 subunit protein in *Resf1* shRNA KD cells (S9D Fig). However, metastasis assays revealed that reduced RPL22 in 6DT1 cells did not significantly alter metastasis (S9E–S9H Fig). Consistent with ribosomal RNA FISH data, KD of *Ubf* (S10C Fig), a transcription factor for 45S ribosomal precursor RNA, did not significantly reduce the expression of all 4 rRNA subunits (S10A Fig). *Ubf* KD also did not change the ability of tumor cells to form lung metastases (S10D–S10F Fig). Taken together, these results suggest that the *Resf1*-mediated metastasis phenotype is not associated with large alterations in ribosomal biogenesis or translational efficiency.

### RESF1 metastatic phenotype is not associated with epigenetic silencing of endogenous retroviruses

RESF1 was recently found to interact with the histone lysine methyltransferase SETDB1 to epigenetically repress endogenous retroviral elements in embryonic stem cells [28]. To determine whether suppression of *Resf1* altered the expression of endogenous retroviral elements in breast cancer cell lines, qRT-PCR analysis was performed. No differences in the expression of any of the endogenous elements were observed (S11A Fig). Furthermore, global levels of H3K9me3 were unchanged (S11B Fig). To more directly test whether the RESF1 metastatic phenotype was mediated by SETDB1, metastasis assays were performed with *Setdb1* shRNA KD 6DT1 cells (S11C Fig). Although a significant reduction of metastasis was observed in the sh975 cell lines (S11D Fig), this result was not observed for the other two shRNAs utilized (S11E Fig). Together, these data suggest that the metastatic phenotype observed upon *Resf1* KD is not related to the interaction of RESF1 with SETDB1 or the reactivation of endogenous retroviruses.

### RESF1 depletion suppresses epithelial-to-mesenchymal transcriptional programs

**Fig 8:**
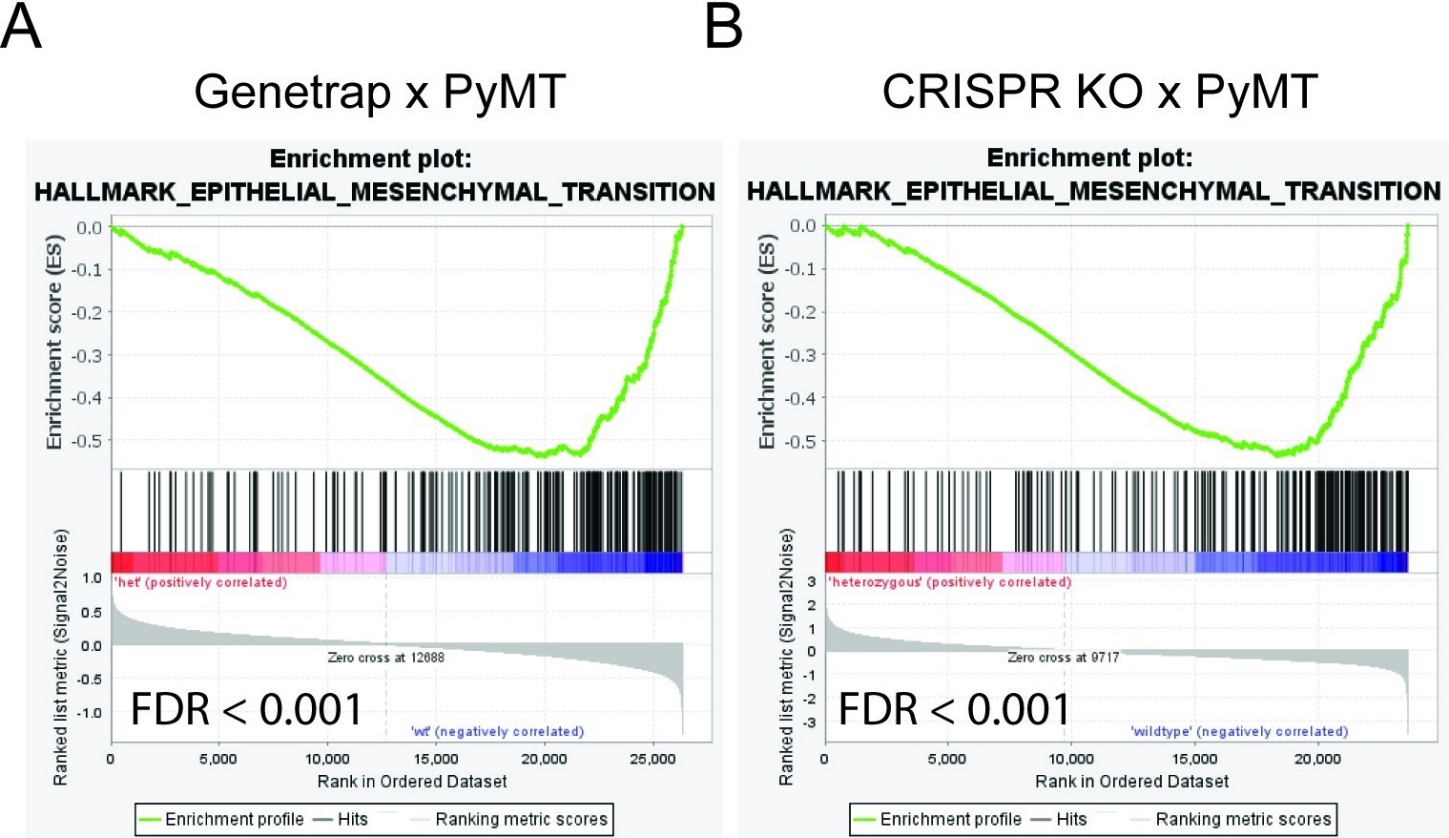
*Resf1* depletion enhances the expression of *Myc* targets. (A-D) GSEA analysis snapshots of MYC-target pathways that are upregulated in 6DT1 shRNA KD, 4T1 CRISPR KO, HEK293T siRNA, and MDA-MB-231 shRNA KD. (E) BioTAP XL analysis of the association between RESF1 and *MYC* exons.

RESF1 has also been implicated in mouse embryonic stem cell self-renewal and germ cell specification [29]. Cellular plasticity and stem cells are linked to epithelial-to-mesenchymal transition (EMT) [30], which is a process implicated in metastasis where cells lose expression of epithelial markers and gain mesenchymal and stem-like phenotypes. To investigate the potential role of cellular plasticity, tumor sphere analysis, which is thought to measure stem-like capacity in cell lines, was performed. However, growth in 3D culture was not significantly and consistently different for both mouse and human breast cancer KD and KO cell lines (4T1, 6DT1, Met1, and MDA-MB-231; Fig 7E and 7F), arguing against an increase in stem-like properties. However, given that opposite results were observed between the allograft and autochthonous GEM model metastasis assays and that previous work from our laboratory has demonstrated that tumor cells rapidly acquire permanent mesenchymal-like transcriptional programs after *in vitro* culture [31], GSEA analysis of the autochthonous primary tumors was examined. In contrast to the *in vitro* models, GSEA analysis of both the KO x PyMT and Genetrap x PyMT tumors demonstrated a highly significant suppression (FDR < 0.001) of EMT-associated transcriptional programs in the autochthonous setting (Fig 8A and 8B). Mesenchymal breast cancer cells are thought to be non-proliferative. Thus, the *in vivo* suppression of the EMT transcriptional program in the *Resf1* depleted autochthonous tumors is consistent with the increased tumor growth and decreased tumor latency. The precise mechanism for this phenomenon, however, has yet to be determined.

In summary, here we present evidence that RESF1 is a chromatin-associated nucleoplasmic protein that functions as a tumor suppressor in ER- breast cancers. Depletion of RESF1 results in increased tumor growth and metastasis, potentially through suppression of the EMT pathway. RESF1 is enriched on exons of actively transcribed genes that contain 1 or more predicted DNA G4 quadruplex on both the template and non-template strand, implying an important role in transcriptional elongation. However, the precise mechanism by which RESF1 alters metastatic potential remains unclear at present. Further investigations will be required to elucidate the function of this large, unstructured protein and its role in cancer.

## Discussion

Among breast cancer subtypes, the ER- subtype, which comprises about 15% of all cases, is associated with the most unfavorable outcomes [1]. Patients with this subtype experience relapse and progression to metastatic disease, typically within the first few years following the diagnosis of the primary tumor. In contrast to the more prevalent Luminal or HER2-amplified tumors, there are currently no available targeted therapies for ER- breast cancer [32]. This limitation confines the treatment options for these patients to standard chemotherapy and/or radiation therapy [32]. Consequently, further investigations into the advanced stages of ER- breast cancer, particularly its progression to overt metastatic disease, is essential to pinpoint potential targets to reduce the mortality associated with this breast cancer subtype.

Here, we have exploited the genomes of two different strains of mice that exhibit significantly different tumor phenotypes[8] to identify and characterize RESF1 as a novel tumor suppressor for ER- breast cancer. Previous genetic analysis of an [FVB/NJ(MOLF/EiJ x PyMT)]N2 backcross mapping population revealed an association of 5 genes whose expression correlated with the metastatic burden on the distal end of mouse chromosome 6. Subsequent analysis validated the nearby circadian rhythm gene, *Arntl2* [8], as a bona fide metastasis susceptibility gene. In this study, *Resf1* was selected for analysis because it was significantly associated with metastatic disease after quantitative trait locus analysis and is located near the maximum of the genetic susceptibility association peak. Unpublished studies have not supported the candidate gene, *Etfbkmt*, as a metastasis-associated factor. The remaining 2 candidate genes in the genetically defined region, *Fgfr1op2*, and *Slco1a5*, have not yet been evaluated for their role in metastatic disease.

As described above, *Resf1* is a poorly characterized gene, producing a 1521 amino acid product that is encoded primarily by a single exon. The gene and the intron-exon structure are conserved across species; however, the primary amino acid sequence shows substantially more sequence divergence between species than the average gene (44% identities compared to an average of ~85% for mouse versus human). In addition, RESF1 contains only a single large protein domain of unknown function, and structural algorithms suggest the protein is highly unstructured. Since disordered protein domains are thought to be stabilized by post-translational modifications or interactions with binding partners [33] [34], RESF1’s secondary or tertiary structure may be more critical to protein function than the primary sequence. Further analysis will be required to investigate the validity of this hypothesis.

Unexpectedly, attempts to validate *Resf1* specifically as a metastasis susceptibility gene revealed conflicting results. Expression analysis in the human breast cancer METABRIC data set and in the autochthonous PyMT x MOLF/EiJ backcross tumor samples indicated that lower expression of *Resf1* was associated with worse outcomes. This interpretation was supported by the experimental cross between the *Resf1* gene trap and the PyMT mouse, where animals with reduced *Resf1* expression had higher incidence of metastatic disease and increased numbers of pulmonary metastases. The orthotopic allograft spontaneous metastasis assays in multiple cell lines from two different species, however, suggested that decreased *Resf1* was associated with suppression of metastatic disease. Because this gene-trap animal has not been extensively characterized and single-gene tumor expression studies can be confounded by amplifications or deletions that include additional genes that might influence the phenotype, we incorporated a second CRISPR-mediated *Resf1* KO animal into the study. Breeding this animal to the PyMT tumor model replicated the results from the gene trap experiment and the mouse and tumor *Resf1* gene expression prognosis analysis. The concordance of the results within the allograft or the GEMM autochthonous tumor samples and models suggests that *Resf1* may have diametrically opposite effects on primary tumor growth and metastatic disease, as has been observed for TGFβ. Under this hypothesis, increased metastasis observed in the autochthonous models may result from increased tumor burden providing a greater pool of potentially metastatic clones. Alternatively, the decreased metastasis observed in the allograft models may result from an interaction of RESF1 depletion and a tissue culture-induced adaptation in the tumor cells. Additional studies will be necessary to resolve these possibilities.

Despite the concerns regarding the physiological validity of cell line-based models for investigations into the role of RESF1 in metastatic disease, we initiated a series of mechanistic-based studies to identify potential cell line-based RESF1 metastasis-associated pathways or mechanisms that might be subsequently investigated or validated *in vivo* models. A previous study [35] identified RESF1 as a minor histocompatibility antigen that could be exploited to prevent graft-versus-host disease. To determine if the metastatic mechanism of RESF1 is immune-related, we performed orthotopic fat pad assays in nu/nu mice. As described above, shRNA suppression of *RESF1* in MDA-MB-231 cells implanted into nude mice significantly reduced metastatic disease. Therefore, while RESF1 may function as a minor histocompatibility antigen in tumor-graft rejection, it does not appear to play a significant role in metastatic progression, at least in the cell line-based orthotopic transplant spontaneous metastasis models. Furthermore, the MDA-MB-231 xeno-transplantation experiments into immune-compromised mice suggest that the effect of RESF1 is independent of the adaptive immune system.

To further investigate the role of *Resf1* in metastasis, we conducted a series of studies based on previously published research findings. Ritter et al. [27] found that loss of *Resf1* increases recombinant protein production, but we observed no significant impact on protein production, mTOR pathway activation, or ribosome biogenesis in *Resf1* KO or KD metastatic cell lines. While Fukuda et al. [28] showed that RESF1 regulates endogenous retroviral element silencing along with SETDB1 in mouse embryonic stem cells, retroviral transcript and global H3K9me3 levels were unaffected by RESF1 loss in metastatic cell lines. Moreover, direct targeting of *Setdb1* did not influence metastatic capacity. Lastly, Vojtek and Chambers [29] highlighted RESF1’s impact on embryonic stem cell self-renewal and its interaction with pluripotency transcription factors. BioTAP XL data confirmed the binding of RESF1 to the promoter of the pluripotency transcription factor *Klf4*, and changes in *Vimentin* were observed in *Resf1* KO cells. However, the variable outcomes in sphere assays among mouse and human breast cancer cell lines suggest potential cell line-specific effects of RESF1 in stemness, challenging the notion that RESF1’s previously reported functions directly drive its metastatic phenotype.

*RESF1* has been previously identified as a potential tumor suppressor [18] and as a susceptibility locus for early-onset coronary artery disease in Japanese patients [36]. The potential role of *RESF1* as a tumor suppressor was identified by an analysis of homozygous deletions in 2218 primary tumors across 12 different human cancer types. Our *in vivo* data is consistent with this published report. Intriguingly, our RNA-seq data suggest potential opposing roles at different sites, with higher *Resf1* expression levels in metastases compared to primary tumors in FVB/PyMT mice. These opposing roles at the primary and secondary sites are similar to what is seen for TGFβ, which can be either a tumor suppressor or metastasis-enhancing molecule [37]. The association of *RESF1* with cardiovascular disease was identified in an exome-wide association study of 1,482 patients with cardiovascular disease and 5,774 healthy controls. Neither study, however, included any mechanistic analysis of this gene, so the potential overlap of these phenotypes with a role in breast cancer metastasis is currently unknown.

Protein structure and domain homology analysis, protein-protein interaction, and cellular localization studies are often employed to elucidate protein function. However, existing data for RESF1 are either unavailable or conflicting. For example, previous studies [28,29] reported that RESF1 localizes to the nucleus. In contrast, the Human Protein Atlas Project generated antibodies from two non-overlapping antigens that, when used for immunofluorescent staining, detect RESF1 primarily in the nucleolus. Here, we performed transient transfections to express epitope-tagged RESF1 in HEK293 cells and detected RESF1 in the nucleoplasm. Since transient transfections can nonspecifically target other compartments in the cell, attempts were made to stably transduce *Resf1* constructs into six different mouse mammary tumor cell lines. Evidence of stable transduction was observed in only one cell line, at a level insufficiently low for further molecular analyses (S12A and S12B Fig), suggesting there is an upper limit for RESF1 for cell viability in tissue culture conditions. The generation and validation of the mouse western blot compatible antibody combined with subcellular fractionation suggest that RESF1 is primarily a nucleoplasmic protein, though staining of the dTAG CRISPR knockin cells suggests that RESF1 is capable of translocating into the nucleolus and nuclear speckle under some circumstances.

As previously mentioned, although RESF1 is a large protein, primary amino acid sequence analysis identified only a single domain of unknown function. Moreover, although the gene structure is conserved across species, the amino acid sequence and size of the resulting protein are highly variable, with few conserved regions, most of which appear to be concentrated in the carboxy third of the protein. Furthermore, structural analysis suggests that the majority of the protein is likely disordered, with only a conserved single high-confidence alpha helix identified by Alphafold in the N-terminal third. Taken together, these data suggest that the primary sequence of RESF1 is less important than the inherent disorder that separates the conserved domains. Inherently disordered proteins are thought to mediate function partially through post-translational modification-mediated protein-protein interactions. Examination of the Phosphosite Plus website [38] reveals the presence of conserved phosphorylated, consistent with this possibility. However, to date, repeated efforts to identify protein interaction partners have been unsuccessful, suggesting that RESF1 protein interactions may be transient and/or of low avidity that is not amenable to standard immunoprecipitation mass spectrometry methods. Alternative methods such as proximity labeling will likely be necessary to gain a better understanding of the molecular partners of RESF1.

Our current understanding of RESF1 function is primarily based on the BioTAP XL analysis of the human ortholog RESF1, deposited by the Elledge laboratory in the publicly available GEO database. The DNA proximity labeling conducted in HEK293 cells revealed RESF1’s association with ribosomal RNA repeats, noncoding RNAs, and exons of protein-coding genes, particularly enriched in ribosomal protein components. Despite these associations, our RESF1 depletion results do not indicate global differences in protein translation and ribosomal biogenesis in RESF1-depleted cells. It is possible that alterations in translation may be specific to specific mRNAs, such as the construct used in the Ritter et al. study [27], or that the alterations are below the resolution of the assays used here. Furthermore, our investigations into the direct effects of ribosomal biogenesis on metastasis, through depletion of the ribosomal protein component *Rpl22* or rRNA transcription factor *Ubf*, did not result in alterations in tumor phenotypes. Together, these findings suggest that while RESF1 is significantly associated with the ribosomal biogenesis pathway, it may not play a major role in RESF1’s tumor suppressor functions.

Further detailed examination of Resf1 binding to DNA sequences revealed the association between sequences exhibiting high GC content. Motif analysis suggested the presence of sequences associated with the formation of DNA G4 quadruplexes, known knot-like structures associated with various cellular processes, either on the template or non-template strand [39]. In cancer, stabilized or induced G4s can lead to telomere maintenance issues [40,41,42], reduced oncogene expression [43,44,45], genome instability [21,46,47,48,49], or apoptosis [50,51,52]. Breast cancer is highly heterogeneous, and a study identified the correlation between DNA G4 quadruplexes and increased intratumor heterogeneity [53]. DNA G4s are commonly found on the non-template strand across exons. However, analysis of the RESF1-associated sites revealed that 96.6% of the top 1000 sites had predicted DNA G4 quadruplexes on both strands using the QGRS prediction algorithm. Moreover, 95.5% of the top RESF1-associated sites contained 2 or more predicted DNA G4 quadruplexes on at least one strand, suggesting RESF1 specifically associates with sequences capable of forming complex secondary structures formed by multiple DNA G4 quadruplexes. This is exemplified by analysis of the RPL27, where RESF1 showed significant association only across exon 3, which contains three DNA G4s distributed on both strands, while the other exons have single predicted DNA G4 quadruplexes, all restricted to the non-template strand.

Similar modest changes in overall transcription were observed in the global analysis of the RNA-seq data. DNA G4 quadruplexes have complex effects on transcription, depending on whether they exist on the template or non-template strand [43]. The formation of R-loops, RNA:DNA three-stranded hybrid structures occurring in highly expressed genes, in conjunction with DNA G4 quadruplexes, can significantly impact transcriptional output [23,54]. Despite RESF1’s robust association with a high abundance of actively transcribed genes, depletion of RESF1 surprisingly yields relatively modest effects on the transcriptome, with changes typically falling in the range of 1.2–1.5 fold. These modest effects are observed for transcripts for all three polymerases, including rRNA (Pol I), b-Actin (Pol II), and 7S RNA (Pol III). These effects argue against the major role of RESF1 in controlling global transcription.

Finally, while RNA-seq analysis revealed relatively modest transcriptional effects upon RESF1 depletion, the *in vivo* expression data pointed towards the suppression of EMT-associated pathways following RESF1 loss (summarized in Table 1). Current thought suggests that breast cancer cells that acquire mesenchymal transcription patterns are non-proliferative and that cells must revert to a more epithelial state prior to expansion. Reduced EMT in RESF1-depleted tumors would therefore favor increased proliferation, as is observed. Current thought would also suggest that these tumors would be less metastatic, since EMT is thought to be an important intermediate in tumor progression. Reduced metastatic capacity of the allograft models would be consistent with this interpretation. The increased metastasis in the autochthonous GEM models would be inconsistent with this interpretation. However, it is possible that the increased metastatic capacity of the GEM models may be due to an increased pool of potential metastatic subclones in the primary tumor due to more rapid tumor growth. Clarifying this, as well as many additional mechanistic questions regarding RESF1, will require significant amounts of additional future effort.

**Table 1:** Paradoxical results of allograft and autochthonous models. A summary of the effects of tumor burden, pulmonary metastasis, and EMT-suppression in allograft and autochthonous models.

Model System	Effect on Tumor Burden	Effect on Pulmonary Metastasis	Effect on EMT-Associated Transcriptional Programs
**Allograft**	**No Difference**	**Reduced**	**Non-significant**
**Autochthonous**	**Increased**	**Increased**	**Significant Suppression**

## Materials and methods

### Ethics statement

All animal studies were performed under Animal Study Protocols LPG-002 and LCBG-004, approved by the Bethesda NCI Animal Care and Use Committee.

### MOLF/EiJ backcross and identification of candidate genes

The FVB/NJ (RRID:IMSR_JAX:001800) x [MOLF/EiJ (RRID:MGI:3487124) x MMTV-PyMT/FVB (RRID:MGI:3032640)] cross resulted in 171 N2 backcross animals as previously described in [55]. N2 backcross animals were genotyped using the Center for Inherited Disease Research (www.cidr.jhmi.edu). QTL analysis mapping was conducted by utilizing the R/QTL software with the J/QTL interface [56]. QTL peaks were deemed significant when their p-values fell below 0.05 following correction for genome-wide significance through 10,000 permutations [8]. To identify potential candidate metastasis susceptibility genes, Affymetrix analysis was performed on tumors from the MOLF backcross as previously described [8] and candidate genes prioritized for analysis based on proximity to the LOD score peak.

### Cell lines and culture methods

Mouse and human cell lines were cultured for several experiments. Mouse 6DT1 (RRID:CVCL_C8PZ), 4T1 (RRID:CVCL_0125), and Mvt1 (RRID:CVCL_C8Q2), MDA-MB-231 (RRID:CVCL_0062) cells (generously provided by Dr. Lalage Wakefield, NCI) and human HEK293T (RRID:CVCL_0045) cells were cultured in full DMEM (Gibco CAT#: 10313039, 10% FBS, 1% penicillin-streptomycin (P/S), 1% glutamine). Cells were incubated at 37°C with 5% CO_2_ in tissue culture flasks or dishes. Stable KD cell lines were cultured with various selection antibiotics described below.

### Virus production for lentiviral stable knockdown

TLC lentiviral shRNA constructs against *Resf1* were obtained from Dharmacon. shRNAs targeting the mouse *Resf1* gene, sh-*Resf1* H4 (5’-TTTCGATTTGTAACATAGGTC-3’) and sh-*Resf1* H6 (5’-AAACAACCAAGTTACCTCAGG-3’) were used for *Resf1* KD in mouse cell lines. For *RESF1* KD in human cell lines, shRNAs targeting the human *RESF1* gene, sh-*RESF1* 565 (5’-GAATACAAACTCTGTGGAAGA-3’) and sh-*RESF1* G5 (5’-GATGGATTTGAGATGCTACAA-3’) were used.

For lentivirus production, 1 μg of shRNA-containing plasmids and 1 μg of viral packaging plasmids, pMD2.G (250 ng) (Addgene plasmid #12259) and psPAX2 (750 ng) (Addgene plasmid #12260) (both were obtained as a gift from the Trono lab), were transfected into the human 293T cell line (Thermo Fisher Scientific) using X-tremeGene 9 DNA transfection reagent (Roche). 48 hours post-transfection, virus-containing supernatant was passed through a 45 μm filter to obtain viral particles, which were transferred to human or mouse breast cancer cell lines. 24 hours post-transduction, the viral media was replaced with fresh 10% DMEM. Finally, after 24 hours, the cells were selected with 10 μg/ml puromycin (Sigma) or 5 μg/ml blasticidin (Gibco) in complete DMEM.

### Orthotopic mouse injections

Female virgin FVB/NJ mice were obtained from The Jackson Laboratory at ~6 weeks of age and housed in the NCI Animal Facility for 2 weeks to acclimate before injections at ~8–9 weeks of age. Virgin female athymic NU/J (RRID:IMSR_JAX:002019) mice were obtained from The Jackson Laboratory at the same age but were not injected until ~18 weeks of age.

For FVB/NJ mice, 6DT1 and Mvt1 cells were plated in P/S and selective antibiotic-free media two days before injections. For NU/J mice, MDA-MB-231 cells were plated in the same manner. On the second day, cells were lifted with 0.05% trypsin, washed 3 times in PBS, counted, and resuspended in room temperature PBS to a concentration of 1 x 10^5^ cells per 100 μl. For spontaneous metastasis assays, 100 μl of solution containing 1 x 10^5^ cells were surgically injected into the 4th mammary fat pad of the mice. Mice were euthanized after 28–30 days using an intraperitoneal administration of Avertin followed by cervical dislocation. Primary tumors were removed and weighed, lungs were removed, and surface metastases were counted then placed in formalin for 24 hours followed by 70% ethanol. All singular metastasis assay dissections were conducted by a single investigator after being blinded.

### FVB/NJ x C57BL/6J *Resf1* knockout mice

For semi-tumor non-autonomous investigation of *Resf1*, male *Resf1* heterozygous C57BL/6J (C57BL/6NCrl-2810474O19Rik^em1(IMPC)Mbp/Mmucd^: RRID:MMRRC_048846-UCD) mice carrying a CRISPR-Cas9 mediated deletion of exon 4 were bred with 11-week-old WT female FVB/NJ mice. To genotype the F1 generation, we followed the protocol provided by the Knockout Mouse Project at UC Davis, where the C57BL/6J *Resf1* KO parental mice were originally obtained. Spontaneous metastasis assays were performed on heterozygous F1 females as previously described. Primary tumors and lungs were removed and analyzed as previously described.

For the gene trap hypomorph mouse experiment, the mouse strain used for this research project B6(129S)-Et(EGFP/cre)^16255Rdav/Mmmh^, RRID:MMRRC_034574-MU, was obtained from the Mutant Mouse Resource and Research Center (MMRRC) at University of Missouri, an NIH-funded strain repository, and was donated to the MMRRC by Ronald L. Davis, Ph.D., The Scripps Research Institute and Paul Overbeek, Ph.D., Baylor College of Medicine.

### MMTV-PyMT/FVB x C57BL/6J *Resf1* knockout mice

For a true tumor non-autonomous investigation of *Resf1*, female *Resf1* heterozygous mice were crossed with male MMTV-PyMT/FVB mice. All mice in the F1 generation were genotyped for both the PyMT transgene and *Resf1* zygosity. Females that were WT and heterozygous for *Resf1* and containing the PyMT transgene were maintained until 120 days when they were euthanized. Dissections were conducted by a single investigator after being blinded to the genotype of the mice. Primary tumors were weighed, and surface pulmonary metastases were counted.

### CRISPR knockout cell lines

Single-guided RNA (sgRNA) against the *Resf1* coding region was designed using the CRISPick program [57] (https://portals.broadinstitute.org/gppx/crispick/public). Three sgRNAs targeting the different regions of the largest exon (exon 3) were selected: sgRNA1 5’-TATCTCATAATCCCGACGGA-3’, sgRNA 2 5’-TCGGGAAACTGATATGTTCA-3’, and sgRNA 3 5’-TGTGACAAGTTGGCGGAACC-3’. sgRNAs were annealed and ligated into the LentiCRISPRV2-GFP vector (obtained as a gift from Dr. Ji Luo Lab, NCI). To create stable CRISPR KO cell lines, 1 x 10^6^ 293T cells were plated in 6 cm dishes in P/S-free 10% FBS DMEM media 24 hours before transfection. Cells were transfected with 1 μg of sgRNA and 1 μg of viral packaging plasmids (250 ng pMD2.G and 750 ng psPAX2), as described above, using 6 μl of Xtreme Gene 9 transfection reagent (Roche). After 24 hours of transfection, media was replaced with fresh 10% DMEM, supplemented with 1% P/S and 1% Glutamine, for another 24 hours. Virus-containing supernatant was then passed through a 45 μm filter to obtain viral particles, which were transferred to 50,000 4T1 and 6DT1 cells. 24 hours post-transduction, the viral media was replaced with fresh 10% DMEM. Heterogeneous 4T1 and 6DT1 KO cells were FACS-sorted by GFP fluorescence. A total of 1000 cells were sorted and single cell clones were manually plated into 96-well plates. Clones were allowed to grow until they reached confluence. CRISPR KO of each clone was confirmed by genomic DNA PCR and Sanger sequencing. KO was further validated by western blot using a custom antibody generated against mouse RESF1 protein (GeneScript) at 1:000 dilution.

### RESF1-dTAG-HA construct

RESF1-dTAG knockin vector was generated using the previously described protocol[58]. C-terminal targeting guide RNA was designed using the CRISPick webpage, and sgRNA oligonucleotides were obtained from IDT, annealed, and ligated in the BbsI restriction site of pX330A-1×2 (Addgene, #58766) plasmid to create the pX330A-1x2-cRESF1 vector. The PITCh sgRNA from the pX330S-2-PITCh (Addgene #63670) vector was then inserted into the pX330A-1x2-cRESF1 vector using Golden Gate Assembly (New England Biolab). To generate the donor vector containing dTAG-2xHA sequences, pCRIS-PITChv2-dTAG-Puro (RESF1) (Addgene, #91796) vector was utilized, serving as a template for PCR amplification of the dTAG-2xHA sequence. The PCR product, which included homology arms flanking the sgRNA region and replaced the RESF1 homology arm, was Gibson cloned (NEBuilder Hifi DNA assembly kit, NEB) into the same vector after digestion with MluI restriction enzyme.

To generate the RESF1 dTAG knockin cell line, human 293FT cells were grown on 10cm dishes and transfected with sgRNA-containing vector (pX330A-1x2-cRESF1/PITCh) and donor vector using Xtremegene 9 transfection reagent (Roche). After 72 hours, positive clones with the c-terminal RESF1 dTAG knockin were selected by treating with 1 μg/ml puromycin for 7 days, followed by single-cell cloning. PCR genotyping confirmed the presence of the RESF1 knockin.

### RNA isolation, qRT-PCR, and RNA-seq

For qRT-PCR, cells were seeded at equal density on tissue culture dishes 24 hours before RNA isolation. After 24 hours, media was aspirated and TriPure isolation reagent (Roche) was added to the dish to collect cells before purification. The concentration of pure RNA was measured using the DeNovix DS-11 Spectrophotometer before being reverse transcribed at equal concentration using iScript (BioRad), following the manufacturer’s recommended protocol. Quantitative real-time PCR (qRT-PCR) was performed using Veriquest SYBR green PCR master mix (Applied Biosystems). mRNA expression was measured by the threshold cycle. Normalization of target mRNA counts was normalized to Peptidylprolyl isomerase (*Ppib*) for both human and mouse. The Ct of the normalization gene was subtracted from the Ct of the target genes (ΔCt). Expression levels of the target genes were calculated using the equation: Expression = 2^-(ΔCt)*1000 and compared to the control. Controls were normalized to 1 and experimental targets were relatively compared. Primer sequences are provided in the supplementary information (S1 Table).

For RNA-seq, cells were seeded at equal density in triplicate per condition. After 24 hours, media was aspirated and TriPure isolation reagent was added to the dish to collect cells before centrifugation to separate RNA and organic phases. After separation, the RNA phase was transferred to a new tube and isopropanol was added to the RNA to precipitate. The RNA isopropanol precipitation sample mixture was added to a Qiagen RNeasy Spin column for purification using the Qiagen RNeasy kit. Samples were analyzed by the Agilent 2200 TapeStation electrophoresis system. All samples used for RNA-seq had an RNA integrity number (RIN) score greater than 7 and were sent to the Illumina Sequencing Core at the Frederick National Labs for sequencing.

### Protein extraction

For protein isolation, cells were seeded at equal densities on tissue culture dishes. After 24–48 hours, media was aspirated from the dish and cells were washed once with PBS. For cell lysis, Golden Lysis Buffer (20 mM Tris pH 8.0, 400 mM NaCl, 5 mM EDTA, 1 mM EGTA, 10 mM NaF, 1 mM Na pyrophosphate, 1% Triton X-100, 10% glycerol, Complete EDTA-free protease inhibitor cocktail (Roche), and phosphatase inhibitor (Sigma)) was added to the dish, which was incubated on ice for 20 minutes, after which time cells were scraped off and added to an Eppendorf tube. Proteins were extracted by centrifugation. Protein concentration was measured using the Pierce BCA Protein Assay Kit and measured at 560 nm wavelength on a Versamax spectrophotometer.

### Western blotting

Equal amounts of protein lysate previously measured to 20 μg had proportional amounts of NuPage Reducing agent and NuPage LDS Sample Buffer (Invitrogen) added to each sample. Proteins were denatured by boiling for 5 minutes at 95°C before being loaded onto NuPage Bis-Tris or Tris-Acetate gels with appropriate buffer (MOPS buffer for Bis-Tris gels or Tris-Acetate buffer for Tris-Acetate gels) to separate the proteins in the gel during the run cycle. Gels were then removed from the cassettes and transferred to PVDF membranes (Millipore) using a wet transfer method. Membranes were stained with Ponceau, then blocked in 5% milk in TBST (TBS + Tween) for one hour before adding a primary antibody diluted in fresh 5% milk for overnight incubation at 4°C. Membranes were then washed 3 times with TBST for 10 minutes each before adding a secondary antibody diluted in 5% milk for 1 hour at room temperature. Membranes were washed 3 times again with TBST. Before imaging, ECL Prime Western Blotting Detection Reagent (GE Healthcare) was added for 5 minutes for activation. Membranes were imaged with ImageQuant 800 OD (Amersham). Densitometry analysis was performed using ImageJ.

The following primary antibodies were used: mouse anti-Actin (1:10,000; Abcam), rabbit anti-RESF1 (1:1000; custom), and rabbit anti-KIAA1551 15622 (1:1000; Sigma). The following secondary antibodies were used: goat anti-rabbit (1:5000; Santa Cruz) and goat anti-mouse (1:10,000; GE Healthcare).

### Nuclear co-immunoprecipitation

Co-immunoprecipitations were performed using the Active Motif Nuclear Complex Co-IP Kit following the manufacturer’s recommended nuclear fractionation protocol. 24–48 hours before nuclear isolation, cells were seeded at equal densities on tissue culture dishes. Nuclear lysates were added to a 2 mL low retention tube at equal concentrations along with the appropriate kit buffer and 2 μg of the appropriate antibody, then incubated overnight rotating at 4°C. The next day, Protein G Dynabeads (Invitrogen) capture beads were washed 2 times with the kit wash buffer, then 50 μl of beads were added to each sample and placed on the rotator for 1 hour rotating at 4°C. After incubation, samples with beads were washed 3 times with kit buffer. 2X NuPage buffer and reducing agent buffer were added to each sample at an appropriate amount before boiling at 95°C for 5 minutes. Samples were then loaded onto PAGE gels for western blot as described.

### Ethinyl uridine (EU) pulse

Cells were seeded at equal densities on 12-mm glass coverslips (Electron Microscopy Sciences, catalog #71887–04) that were placed on a plate. After 24–48 hours, cells were incubated for 30 minutes with EU from a Click-iT RNA Imaging Kit (Invitrogen). After incubation, cells were fixed and processed through the kit protocol. After Click chemistry was utilized to add Alexa Fluor 594 to EU, DAPI was added, and then cells were washed twice with PBS. Cells were mounted on slides with ProLong Glass Antifade Mountant (Invitrogen). Confocal images were taken using a Zeiss LSM 780 in the NCI Microscopy Core Facility.

### O-propargyl-puromycin (OPP) pulse

Cells were seeded at equal densities on 12-mm glass coverslips (Electron Microscopy Sciences, catalog #71887–04) that were placed on a plate. After 24–48 hours, cells were incubated for 30 minutes with OPP from a Click-iT Protein Imaging Kit (Invitrogen). After incubation, cells were fixed and processed through the kit protocol. After Click chemistry was utilized to add Alexa Fluor 594 to EU, DAPI was added, and then cells were washed twice with PBS. Cells were mounted on slides with ProLong Glass Antifade Mountant (Invitrogen). Confocal images were taken using a Zeiss LSM 780.

### Immunofluorescence staining

Cells were seeded at equal densities on 12-mm glass coverslips (Electron Microscopy Sciences, catalog #71887–04) that were placed on a 12-well plate. After 24–48 hours, media was aspirated from the well, washed 3 times with PBS then fixed with 4% paraformaldehyde (PFA) for 20 minutes. PFA was removed, and then coverslips were washed twice with PBS. After washing, 0.5% Triton X-100 in PBS was added for 5 minutes at room temperature to permeabilize the cells. After permeabilization, cells were washed 3 times with PBS, and 5% BSA in PBS was added for 1 hour for blocking. After blocking, the primary antibody diluted in 5% BSA was added to the coverslips and incubated overnight at 4°C. After incubation, coverslips were washed 3 times with PBS before adding diluted secondary antibody in 5% BSA in PBS for 1 hour at room temperature followed by 3 washes with PBS. After washing, DAPI diluted in PBS (1 μg/ml final concentration) was added to the coverslips and incubated for 10 minutes at room temperature. The coverslips were again washed 3 times with PBS before mounting on slides with ProLong Glass Antifade Mountant (Invitrogen). Confocal images were taken using a Zeiss LSM 780.

The following primary antibodies were used: C12orf35 13816 (1:500; Sigma HPA013816), KIAA1551 15622 (1:500; Sigma HPA015622), 186C7 (1:500; GenScript custom antibody), Fibrillarin (1:500; Abcam), NPM1 (1:500; Abcam), SC35 (1:1000; Sigma) and HA-tag (1:200; Cell Signalling). Secondary antibodies used were mouse Alexa Fluor 488 (1:200; Invitrogen), rabbit Alexa Fluor 568 (1:200; Invitrogen), and rabbit Alexa Fluor 647 (1:200, Invitrogen)

### PolyA mRNA FISH

Cells were seeded at equal densities on 12-mm glass coverslips (Electron Microscopy Sciences, catalog #71887–04) that were placed on a 12-well plate. After 24–48 hours, media was aspirated from the well, washed once with PBS then fixed with 4% paraformaldehyde in PBS for 10 minutes at room temperature. Paraformaldehyde was removed, and 100% ice-cold methanol was added to the coverslips to permeabilize the nucleus for 10 minutes. Methanol was removed, and 70% ethanol was added for at least 10 minutes to rehydrate the cells after the fixation steps. 1 M Tris pH 8.0 was then added for 5 minutes, removed, and hybridization buffer (1 mg/ml yeast tRNA, 0.005% BSA, 10% dextran sulfate, 25% deionized formamide, 2X SSC, and 1 ng/μl fluorescent 5’-Cy3-Oligo d(T)50 probe (Gene Link, catalog #26-4322-02)) was added to the coverslips and incubated at 37°C overnight sealed in a plastic bag with wet kimwipes to maintain humidity. After hybridization, coverslips were washed once with 4X SSC for 5 minutes, and again with 2X SSC for 5 minutes. After washing, coverslips were incubated with 1 μg/ml DAPI in 2X SSC and 0.1% Triton X-100 for 15 minutes and washed twice with 2X SSC for 5 minutes each. Coverslips were mounted on glass slides with ProLong Glass Antifade Mountant (Invitrogen). Confocal images were taken using a Zeiss LSM 780.

### RNA FISH

Cells were seeded at equal densities on 12-mm glass coverslips (Electron Microscopy Sciences, catalog #71887–04) that were placed on a 12-well plate. Coverslips were washed once with PBS and fixed in 4% paraformaldehyde in PBS for 10 minutes at room temperature and washed once again with PBS. 70% ethanol was added to the coverslips and incubated at 4°C for 1 hour. Coverslips were washed with 2X SSC with 10% formamide for 5 minutes, then permeabilized with 0.5% Triton X-100 in 2X SSC for 10 minutes at room temperature. Hybridization buffer (10% dextran sulfate, 1 mg/mL yeast tRNA, 10% formamide, 2X SSC, and RNase-free water) with an RNA FISH oligo labeled with a Cy3 fluorophore (1 μl per 100 μl hybridization reaction) was added to each sample and incubated overnight at 30°C sealed in a plastic bag with a few wet kimwipes. After overnight incubation, coverslips were rinsed once and washed with 2X SSC with 10% formamide once for 15 minutes, then washed again for 30 minutes. Coverslips were stained with 5 μg/ml DAPI in 2X SSC and 10% formamide for 30 minutes at room temperature. After DAPI staining, the coverslips were mounted on glass slides with ProLong Glass Antifade Mountant (Invitrogen). Confocal images were taken using a Zeiss LSM 780. Oligo sequences are provided in the supplementary information (S1 Table).

### Tumorsphere assay

Cells were seeded at equal densities on ultra-low attachment 24-well plates (Corning) in MethoCult H4100 mixture containing MammoCult (Cat #05621), MammoCult Proliferation Supplement (Cat #05622), hydrocortisone, and heparin. The plates were mixed for 15 minutes at 4°C rocking to lodge cells into the MethoCult. The plates were then incubated at 37°C undisturbed for 7–12 days. After incubation, spheres were quantified and measured in diameter using Celigo.

### Gel mobility shift assay and circular dichroism

Oligomers (~4.5 μM) forming G4s were incubated in 100 mM potassium chloride, sodium chloride, or lithium chloride in water at 95°C for 5 minutes before cooling to room temperature slowly (~2 h). The reactions were loaded onto 3% agarose gels containing 1X TAE buffer at 100V at room temperature. The signal was detected using BioRad ChemiDoc Imaging System.

Circular dichroism (CD) spectra were recorded on a spectrophotometer using a 1-mm quartz cuvette from Hellma Analytics with a reaction volume of 200 μl. Samples were prepared the same for gel mobility shift assays. An average of three scans were taken for each sample, and the buffer spectrum was subtracted. For equilibrium CD measurements, wavelengths between 230 nm and 315 nm were scanned.

### BioTAP analysis

FASTQ files from the Short Read Archive (PRJNA509912) were downloaded into Partek Flow and based on Phred scores <20 trimmed from the 3’ end. The reads were then aligned to the T2Tv2a or hg38 human genome build using BWA 0.7.17 and peaks called by comparing input and BioTAP XL samples using MACS v 3.0.0a7. Peaks were then quantified and annotated to the T2Tv2a genome using the PartekFlow default settings. Statistical analysis was performed using ANOVA and peaks with an FDR ≤ 0.05 were considered significant. Overlap analysis of the BioTAP XL T2Tv2a peaks was performed by uploading BED files to the UCSC Genome Browser and performing intersection analysis using the UCSC Genome Browser TableBrowser tool. Analysis using the GREAT tool was performed using BED files aligned to the hg38 genome build.

Analysis of the RNA-seq data was performed in Partek Flow by aligning to the T2Tv2a human genome using STAR 2.7.8a using default settings. Reads were normalized by median ratio and differentially expressed transcripts were identified using DESeq2. Transcripts were considered significantly different at an FDR ≤ 0.05.

### Nuclear fractionation analysis

Subnuclear fractionation assays were performed with a Thermo Scientific Subcellular Fractionation Kit for Cultured Cells (cat. No. 78840), following the manufacturers suggested protocol.

### mTOR pathway analysis

mTOR pathway analysis was performed with the Full Moon Biosystems mTOR Phospho Antibody array (cat. No. PMT138) using shScramble and shG5 MDA-MB-231 cell lysates, following the manufacturers recommended protocol.

## Supporting information

S1 Fig Lower expression in identified genes results in poor DMFS. From the N_2_ backcross cohort in Fig 1C, 131 mice were categorized into over and under-expression of *Arntl2* (A), *Resf1* (281047O19Rik) (B), and *Etfbkmt* (4833442J19Rik) (C). Kaplan-Meier curves show worse survival for DMFS when under-expressed.
(TIF)

S2 Fig Upstream single nucleotide variants alter *Resf1* expression in FVB/NJ and MOLF/EiJ. (A) Query of the UCSC BLAT Genome Browser for the 5’ UTR and upstream region of *Resf1* displays DHS peaks (green) in the highlighted yellow area. Included are locations of primers used for PCR and cloning of the promoter enhancer region. (B) Primers used for cloning the upstream region of *Resf1* into pGL4.23 luciferase reporter plasmid.
(TIF)

S3 Fig *Resf1* knockout mice have a higher metastatic burden than WT mice. Control and *Resf1* CRISPR KO mice were crossed with MMTV-PyMT mice to induce spontaneous mammary tumors and pulmonary metastases, which were allowed to grow for 120 days (A-D). (A) Primary mammary fatpad tumors were collected and weighed for WT (n = 21) and KO (n = 17) mice and resulted in significantly larger tumors in KO mice, with p-value calculated by the Mann-Whitney test. (B) Surface lung metastases were counted, resulting in more metastases in KO mice, p-value calculated by the Mann-Whitney test. (C) Normalization of lung metastases per gram tumor to account for larger tumor size remained significant in KO mice, with p-value calculated by the Mann-Whitney test. (D) Metastatic incidence was higher in KO mice compared to control. Orthotopic injection into the 4^th^ mammary fatpad with syngeneic Mvt1 cells into *Resf1* control and hypomorph mice displayed no change in tumor burden or metastasis (E-F). Identical orthotopic injection with syngeneic 6DT1 cells into *Resf1* control and KO mice also displayed no change in tumor burden or metastasis (G-H). (I) A representative image of the genotyped tail and primary tumor for *Resf1* from WT and KO mice shows a loss of a wildtype allele in primary tumors. (J) *Resf1* mRNA expression is higher in lung metastases compared to primary tumors in FVB/NJ x PyMT mice.
(TIF)

S4 Fig *Resf1* is a nucleoplasmic protein. Confocal microscopy images showing nucleolar staining of (A) 15622 antibody, (B) 13816 antibody, and (C) transient transfections of Resf1-mCherry and -V5 in HEK293T cells showing nucleoplasmic staining. (D) Western blot analysis validating custom-generated rabbit antibody raised against mouse *Resf1*. (E) Western blot analysis showing CRISPR KO of *Resf1* in 6DT1 and 4T1 cells. (F) Confocal microscopy images of 4T1 CRISPR KO cells show no differences in 186C7 (4T1), 13816 (4T1), and 15A4 (6DT1) staining between control and sgRNA KO. (G) Subnuclear fractionation western blot analysis of 4T1 and 6DT1 parental cells reveal *Resf1* in the nucleoplasm, validated by the controls (Hsp90, Sp1, H3, and CK18) for each subcellular compartment. (H) Confocal microscopy images of RESF1-dTAG-HA knockin in HEK293T cells, co-stained with either NPM1 or SC35.
(TIF)

S5 Fig RESF1 is associated with transcribed sequences. (A) Gene Ontology analysis showing enrichment in cellular components and (B) biological processes involved with translation, noncoding RNA, and ribosomal biogenesis. (C) Mapping of G4 quadruplexes in cytoplasmic RNA and RPL27 mRNA.
(TIF)

S6 Fig *Resf1* knockdown does not increase nascent RNA production. Confocal microscopy images of (A) 6DT1 shRNA KD and (B) MDA-MB-231 shRNA KD cell lines with a 30-minute EU (ethinyl uridine) pulse.
(TIF)

S7 Fig RESF1 knockdown does not alter the mTOR pathway. (A) mTOR array shows minimal fold changes in various proteins mTOR pathways upon RESF1 KD in MDA-MB-231 cells.
(TIF)

S8 Fig RESF1 knockdown does not alter the unfolded protein response. Western blot analysis of several unfolded protein response (UPR) targets in 6DT1 (blue and purple) and MDA-MB-231 (green and yellow), with and without prior tunicamycin (TM) treatment for activation, and glass (A) or plastic (B) culture surface, shows no consistent change in RESF1 KD cells compared to control.
(TIF)

S9 Fig Ribosomal subunit pathways are decreased in *Resf1* knockdown cells. (A) GO Pathway analysis snapshots of various ribosomal subunit pathways that are decreased in 6DT1 *Resf1* KD cells. (B) The GO Cytosolic Ribosome pathway had many (C) small and large ribosomal proteins decreased in *Resf1* KD cells. (D) Western blot analysis of many of these proteins identified only *Rpl22* decreased at the protein level as well. (E) Western blot analysis of 6DT1 shRNA-mediated *Rpl22* stable KD cells. (F) Weight of primary tumors from 6DT1 Control (scramble), sh14, and sh17 cells orthotopically injected into the 4^th^ mammary fatpad of syngeneic FVB/NJ mice, n = 20 mice per group. (G) Surface pulmonary metastasis in mice from (F). (H) Pulmonary metastases normalized per gram tumor from (F). P-value was calculated by Mann-Whitney test.
(TIF)

S10 Fig Upstream binding factor is not a metastasis modifier. (A) RT-qPCR analysis of 6DT1 *Ubf* control and KD cells reveals a modest but not significant decrease in rRNA subunits and (B) no change in *Resf1* expression levels. (C) Western blot analysis of 6DT1 shRNA-mediated *Ubf* stable KD cells. (D) Weight of primary tumors from 6DT1 Control (scramble), sh90, and sh92 cells orthotopically injected into the 4^th^ mammary fatpad of syngeneic FVB/NJ mice, n = 10 mice per group. (E) Surface pulmonary metastasis in mice from (D). (F) Pulmonary metastases normalized per gram tumor from (D). P-value was calculated by Mann-Whitney test.
(TIF)

S11 Fig *Resf1* knockdown does not alter endogenous retroelements. (A) RT-qPCR analysis of three different families of LINE (L1) elements display relatively no change upon *Resf1* KD in 6DT1 cells. (B) A colorimetric H3K9me3 capture based assay also displayed no significant change in global tr-methyl histone H3K9 between control and RESF1 KD MDA-MB-231 cells, p-value based on Mann-Whitney test. (C) *Setdb1* protein expression in 6DT1 cells shown by western blot, with a transiently transfected overexpression control in 293FT cells. (D) Weight of primary tumors from 6DT1 Control (scramble), 600KD, and 975KD cells orthotopically injected into the 4^th^ mammary fatpad of syngeneic FVB/NJ mice, n = 20 mice per group, combined data from 2 experiments. (E) Surface pulmonary metastases counted from (D). Weight of primary tumors from 6DT1 Control (scramble), 590KD cells orthotopically injected into the 4^th^ mammary fatpad of syngeneic FVB/NJ mice, n = 20 mice per group, combined data from 2 experiments. (G) Surface pulmonary metastases counted from (F). All p-values calculated by Mann-Whitney test.
(TIF)

S12 Fig *Resf1* cannot be stably overexpressed in cell lines. (A) Western blot analysis reveals stable expression of RESF1 in 67NR cell line, as shown with Resf1-mCherry construct. (B) Immunofluorescence analysis showing no specific mCherry signal in 67NR WT and overexpressed cell lines.
(TIF)

S1 Table Oligonucleotide sequences used in this study. (PDF)

## References

[1] SEER [Internet]. [cited 2023 Dec 18]. Cancer of the Breast (Female)—Cancer Stat Facts. Available from: https://seer.cancer.gov/statfacts/html/breast.html

[2] Nowell PC. The clonal evolution of tumor cell populations. Science. 1976 Oct 1;194(4260):23–8. doi: 10.1126/science.959840 959840

[3] Al Bakir M, Huebner A, Martínez-Ruiz C, Grigoriadis K, Watkins TBK, Pich O, et al. The evolution of non-small cell lung cancer metastases in TRACERx. Nature. 2023 Apr;616(7957):534–42. doi: 10.1038/s41586-023-05729-x 37046095 PMC10115651

[4] Chorley BN, Wang X, Campbell MR, Pittman GS, Noureddine MA, Bell DA. Discovery and verification of functional single nucleotide polymorphisms in regulatory genomic regions: Current and developing technologies. Mutat Res. 2008;659(1–2):147–57. doi: 10.1016/j.mrrev.2008.05.001 18565787 PMC2676583

[5] Haraksingh RR, Snyder MP. Impacts of Variation in the Human Genome on Gene Regulation. J Mol Biol. 2013 Nov;425(21):3970–7. doi: 10.1016/j.jmb.2013.07.015 23871684

[6] Guy CT, Cardiff RD, Muller WJ. Induction of mammary tumors by expression of polyomavirus middle T oncogene: a transgenic mouse model for metastatic disease. Mol Cell Biol. 1992 Mar;12(3):954–61. doi: 10.1128/mcb.12.3.954-961.1992 1312220 PMC369527

[7] Lifsted T, Le Voyer T, Williams M, Muller W, Klein-Szanto A, Buetow KH, et al. Identification of inbred mouse strains harboring genetic modifiers of mammary tumor age of onset and metastatic progression. Int J Cancer. 1998;77(4):640–4. doi: 10.1002/(sici)1097-0215(19980812)77:4&lt;640::aid-ijc26&gt;3.0.co;2-8 9679770

[8] Ha NH, Long J, Cai Q, Shu XO, Hunter KW. The Circadian Rhythm Gene Arntl2 Is a Metastasis Susceptibility Gene for Estrogen Receptor-Negative Breast Cancer. PLoS Genet. 2016 Sep;12(9):e1006267. doi: 10.1371/journal.pgen.1006267 27656887 PMC5033489

[9] Bai L, Yang HH, Hu Y, Shukla A, Ha NH, Doran A, et al. An Integrated Genome-Wide Systems Genetics Screen for Breast Cancer Metastasis Susceptibility Genes. PLOS Genet. 2016 Apr 13;12(4):e1005989. doi: 10.1371/journal.pgen.1005989 27074153 PMC4830524

[10] Ringnér M, Fredlund E, Häkkinen J, Borg Å, Staaf J. GOBO: Gene Expression-Based Outcome for Breast Cancer Online. PLOS ONE. 2011 Mar 21;6(3):e17911. doi: 10.1371/journal.pone.0017911 21445301 PMC3061871

[11] Dunham I, Kundaje A, Aldred SF, Collins PJ, Davis CA, Doyle F, et al. An integrated encyclopedia of DNA elements in the human genome. Nature. 2012 Sep;489(7414):57–74. doi: 10.1038/nature11247 22955616 PMC3439153

[12] Hindorff LA, Sethupathy P, Junkins HA, Ramos EM, Mehta JP, Collins FS, et al. Potential etiologic and functional implications of genome-wide association loci for human diseases and traits. Proc Natl Acad Sci. 2009 Jun 9;106(23):9362–7. doi: 10.1073/pnas.0903103106 19474294 PMC2687147

[13] Pei XF, Noble MS, Davoli MA, Rosfjord E, Tilli MT, Furth PA, et al. Explant-cell culture of primary mammary tumors from MMTV-c-Myc transgenic mice. In Vitro Cell Dev Biol Anim. 2004;40(1–2):14–21. doi: 10.1290/1543-706X(2004)40&lt;14:ECOPMT&gt;2.0.CO;2 15180438

[14] Hu Y, Bai L, Geiger T, Goldberger N, Walker RC, Green JE, et al. Genetic Background May Contribute to PAM50 Gene Expression Breast Cancer Subtype Assignments. PLoS ONE. 2013 Aug 28;8(8):e72287. doi: 10.1371/journal.pone.0072287 24015230 PMC3756056

[15] Ross C, Szczepanek K, Lee M, Yang H, Qiu T, Sanford JD, et al. The genomic landscape of metastasis in treatment-naïve breast cancer models. PLoS Genet. 2020 May 28;16(5):e1008743. 32463822 10.1371/journal.pgen.1008743 PMC7282675

[16] Yang Y, Yang HH, Hu Y, Watson PH, Liu H, Geiger TR, et al. Immunocompetent mouse allograft models for development of therapies to target breast cancer metastasis. Oncotarget. 2017 Feb 25;8(19):30621–43. doi: 10.18632/oncotarget.15695 28430642 PMC5458155

[17] Tian M, Schiemann WP. The TGF-beta paradox in human cancer: an update. Future Oncol Lond Engl. 2009 Mar;5(2):259–71. doi: 10.2217/14796694.5.2.259 19284383 PMC2710615

[18] Cheng J, Demeulemeester J, Wedge DC, Vollan HKM, Pitt JJ, Russnes HG, et al. Pan-cancer analysis of homozygous deletions in primary tumours uncovers rare tumour suppressors. Nat Commun. 2017 Oct 31;8:1221. doi: 10.1038/s41467-017-01355-0 29089486 PMC5663922

[19] Alekseyenko AA, McElroy KA, Kang H, Zee BM, Kharchenko PV, Kuroda MI. BioTAP-XL—crosslinking/tandem affinity purification to study DNA targets, RNA and protein components of chromatin associated complexes. Curr Protoc Mol Biol Ed Frederick M Ausubel Al. 2015 Jan 5;109:21.30.1–21.30.32. doi: 10.1002/0471142727.mb2130s109 25559106 PMC4308290

[20] McLean CY, Bristor D, Hiller M, Clarke SL, Schaar BT, Lowe CB, et al. GREAT improves functional interpretation of cis-regulatory regions. Nat Biotechnol. 2010 May;28(5):495–501. doi: 10.1038/nbt.1630 20436461 PMC4840234

[21] Paeschke K, Capra JA, Zakian VA. DNA replication through G-quadruplex motifs is promoted by the S. cerevisiae Pif1 DNA helicase. Cell. 2011 May 27;145(5):678–91. 21620135 10.1016/j.cell.2011.04.015 PMC3129610

[22] Schiavone D, Guilbaud G, Murat P, Papadopoulou C, Sarkies P, Prioleau MN, et al. Determinants of G quadruplex-induced epigenetic instability in REV1-deficient cells. EMBO J. 2014 Nov 3;33(21):2507–20. doi: 10.15252/embj.201488398 25190518 PMC4282387

[23] De Magis A, Manzo SG, Russo M, Marinello J, Morigi R, Sordet O, et al. DNA damage and genome instability by G-quadruplex ligands are mediated by R loops in human cancer cells. Proc Natl Acad Sci U S A. 2019 Jan 15;116(3):816–25. doi: 10.1073/pnas.1810409116 30591567 PMC6338839

[24] Georgakopoulos-Soares I, Parada GE, Wong HY, Medhi R, Furlan G, Munita R, et al. Alternative splicing modulation by G-quadruplexes. Nat Commun. 2022 May 3;13(1):2404. doi: 10.1038/s41467-022-30071-7 35504902 PMC9065059

[25] Zanin I, Ruggiero E, Nicoletto G, Lago S, Maurizio I, Gallina I, et al. Genome-wide mapping of i-motifs reveals their association with transcription regulation in live human cells. Nucleic Acids Res. 2023 Sep 8;51(16):8309–21. doi: 10.1093/nar/gkad626 37528048 PMC10484731

[26] Varshney D, Cuesta SM, Herdy B, Abdullah UB, Tannahill D, Balasubramanian S. RNA G-quadruplex structures control ribosomal protein production. Sci Rep. 2021 Nov 23;11:22735. doi: 10.1038/s41598-021-01847-6 34815422 PMC8611094

[27] Ritter A, Rauschert T, Oertli M, Piehlmaier D, Mantas P, Kuntzelmann G, et al. Disruption of the gene C12orf35 leads to increased productivities in recombinant CHO cell lines. Biotechnol Bioeng. 2016;113(11):2433–42. doi: 10.1002/bit.26009 27183150

[28] Fukuda K, Okuda A, Yusa K, Shinkai Y. A CRISPR knockout screen identifies SETDB1-target retroelement silencing factors in embryonic stem cells. Genome Res. 2018 Jun;28(6):846–58. doi: 10.1101/gr.227280.117 29728365 PMC5991520

[29] Vojtek M, Chambers I. Loss of Resf1 reduces the efficiency of embryonic stem cell self-renewal and germline entry. Life Sci Alliance. 2021 Oct 4;4(12):e202101190. doi: 10.26508/lsa.202101190 34607919 PMC8500223

[30] Mani SA, Guo W, Liao MJ, Eaton ENg, Ayyanan A, Zhou AY, et al. The Epithelial-Mesenchymal Transition Generates Cells with Properties of Stem Cells. Cell. 2008 May 16;133(4):704–15. doi: 10.1016/j.cell.2008.03.027 18485877 PMC2728032

[31] Ross C, Szczepanek K, Lee M, Yang H, Peer CJ, Kindrick J, et al. Metastasis-Specific Gene Expression in Autochthonous and Allograft Mouse Mammary Tumor Models: Stratification and Identification of Targetable Signatures. Mol Cancer Res. 2020 Sep;18(9):1278–89. doi: 10.1158/1541-7786.MCR-20-0046 32513899 PMC7483845

[32] Waks AG, Winer EP. Breast Cancer Treatment: A Review. JAMA. 2019 Jan 22;321(3):288–300. doi: 10.1001/jama.2018.19323 30667505

[33] Trivedi R, Nagarajaram HA. Intrinsically Disordered Proteins: An Overview. Int J Mol Sci. 2022 Nov 14;23(22):14050. doi: 10.3390/ijms232214050 36430530 PMC9693201

[34] Xie H, Vucetic S, Iakoucheva LM, Oldfield CJ, Dunker AK, Obradovic Z, et al. Functional anthology of intrinsic disorder. 3. Ligands, post-translational modifications, and diseases associated with intrinsically disordered proteins. J Proteome Res. 2007 May;6(5):1917–32. doi: 10.1021/pr060394e 17391016 PMC2588348

[35] Oostvogels R, Minnema M, Van Elk M, Spaapen R, Te Raa G, Giovannone B, et al. Towards effective and safe immunotherapy after allogeneic stem cell transplantation: identification of hematopoietic-specific minor histocompatibility antigen UTA2-1. Leukemia. 2012;27(10). doi: 10.1038/leu.2012.277 23079962 PMC3593180

[36] Yamada Y, Yasukochi Y, Kato K, Oguri M, Horibe H, Fujimaki T, et al. Identification of 26 novel loci that confer susceptibility to early-onset coronary artery disease in a Japanese population. Biomed Rep. 2018 Nov;9(5):383–404. doi: 10.3892/br.2018.1152 30402224 PMC6201041

[37] Neel JC, Humbert L, Lebrun JJ. The Dual Role of TGFβ in Human Cancer: From Tumor Suppression to Cancer Metastasis. ISRN Mol Biol. 2012 Dec 24;2012:1–28. 10.5402/2012/381428 PMC4899619 27340590

[38] Hornbeck PV, Chabra I, Kornhauser JM, Skrzypek E, Zhang B. PhosphoSite: A bioinformatics resource dedicated to physiological protein phosphorylation. Proteomics. 2004 Jun;4(6):1551–61. doi: 10.1002/pmic.200300772 15174125

[39] Teng FY, Jiang ZZ, Guo M, Tan XZ, Chen F, Xi XG, et al. G-quadruplex DNA: a novel target for drug design. Cell Mol Life Sci. 2021 Oct 1;78(19):6557–83. doi: 10.1007/s00018-021-03921-8 34459951 PMC11072987

[40] Rodriguez R, Müller S, Yeoman JA, Trentesaux C, Riou JF, Balasubramanian S. A novel small molecule that alters shelterin integrity and triggers a DNA-damage response at telomeres. J Am Chem Soc. 2008 Nov 26;130(47):15758–9. doi: 10.1021/ja805615w 18975896 PMC2746963

[41] Vannier JB, Pavicic-Kaltenbrunner V, Petalcorin MIR, Ding H, Boulton SJ. RTEL1 dismantles T loops and counteracts telomeric G4-DNA to maintain telomere integrity. Cell. 2012 May 11;149(4):795–806. doi: 10.1016/j.cell.2012.03.030 22579284

[42] Salvati E, Leonetti C, Rizzo A, Scarsella M, Mottolese M, Galati R, et al. Telomere damage induced by the G-quadruplex ligand RHPS4 has an antitumor effect. J Clin Invest. 2007 Nov;117(11):3236–47. doi: 10.1172/JCI32461 17932567 PMC2000812

[43] Varshney D, Spiegel J, Zyner K, Tannahill D, Balasubramanian S. The regulation and functions of DNA and RNA G-quadruplexes. Nat Rev Mol Cell Biol. 2020 Aug;21(8):459–74. doi: 10.1038/s41580-020-0236-x 32313204 PMC7115845

[44] Kim N. The Interplay between G-quadruplex and Transcription. Curr Med Chem. 2019 May;26(16):2898–917. doi: 10.2174/0929867325666171229132619 29284393 PMC6026074

[45] Wolfe AL, Singh K, Zhong Y, Drewe P, Rajasekhar VK, Sanghvi VR, et al. RNA G-quadruplexes cause eIF4A-dependent oncogene translation in cancer. Nature. 2014 Sep 4;513(7516):65–70. doi: 10.1038/nature13485 25079319 PMC4492470

[46] Wu Y, Shin-ya K, Brosh RM. FANCJ helicase defective in Fanconia anemia and breast cancer unwinds G-quadruplex DNA to defend genomic stability. Mol Cell Biol. 2008 Jun;28(12):4116–28. doi: 10.1128/MCB.02210-07 18426915 PMC2423121

[47] Wu W, Rokutanda N, Takeuchi J, Lai Y, Maruyama R, Togashi Y, et al. HERC2 Facilitates BLM and WRN Helicase Complex Interaction with RPA to Suppress G-Quadruplex DNA. Cancer Res. 2018 Nov 15;78(22):6371–85. doi: 10.1158/0008-5472.CAN-18-1877 30279242

[48] Wang Y, Yang J, Wild AT, Wu WH, Shah R, Danussi C, et al. G-quadruplex DNA drives genomic instability and represents a targetable molecular abnormality in ATRX-deficient malignant glioma. Nat Commun. 2019 Feb 26;10(1):943. doi: 10.1038/s41467-019-08905-8 30808951 PMC6391399

[49] London TBC, Barber LJ, Mosedale G, Kelly GP, Balasubramanian S, Hickson ID, et al. FANCJ is a structure-specific DNA helicase associated with the maintenance of genomic G/C tracts. J Biol Chem. 2008 Dec 26;283(52):36132–9. doi: 10.1074/jbc.M808152200 18978354 PMC2662291

[50] Rapozzi V, Zorzet S, Zacchigna M, Della Pietra E, Cogoi S, Xodo LE. Anticancer activity of cationic porphyrins in melanoma tumour-bearing mice and mechanistic in vitro studies. Mol Cancer. 2014 Apr 1;13(1):75. doi: 10.1186/1476-4598-13-75 24684778 PMC4021972

[51] Pattanayak R, Barua A, Das A, Chatterjee T, Pathak A, Choudhury P, et al. Porphyrins to restrict progression of pancreatic cancer by stabilizing KRAS G-quadruplex: In silico, in vitro and in vivo validation of anticancer strategy. Eur J Pharm Sci Off J Eur Fed Pharm Sci. 2018 Dec 1;125:39–53. 10.1016/j.ejps.2018.09.011 30223034

[52] Beauvarlet J, Bensadoun P, Darbo E, Labrunie G, Rousseau B, Richard E, et al. Modulation of the ATM/autophagy pathway by a G-quadruplex ligand tips the balance between senescence and apoptosis in cancer cells. Nucleic Acids Res. 2019 Apr 8;47(6):2739–56. doi: 10.1093/nar/gkz095 30759257 PMC6451122

[53] Kosiol N, Juranek S, Brossart P, Heine A, Paeschke K. G-quadruplexes: a promising target for cancer therapy. Mol Cancer. 2021 Feb 25;20(1):40. doi: 10.1186/s12943-021-01328-4 33632214 PMC7905668

[54] Tan J, Lan L. The DNA secondary structures at telomeres and genome instability. Cell Biosci. 2020 Mar 26;10(1):47. doi: 10.1186/s13578-020-00409-z 32257105 PMC7104500

[55] Hunter KW, Broman KW, Voyer TL, Lukes L, Cozma D, Debies MT, et al. Predisposition to efficient mammary tumor metastatic progression is linked to the breast cancer metastasis suppressor gene Brms1. Cancer Res. 2001 Dec 15;61(24):8866–72. 11751410

[56] Smith R, Sheppard K, DiPetrillo K, Churchill G. Quantitative trait locus analysis using J/qtl. Methods Mol Biol Clifton NJ. 2009;573:175–88. doi: 10.1007/978-1-60761-247-6_10 19763928

[57] Nuñez Pedrozo CN, Peralta TM, Olea FD, Locatelli P, Crottogini AJ, Belaich MN, et al. In silico performance analysis of web tools for CRISPRa sgRNA design in human genes. Comput Struct Biotechnol J. 2022;20:3779–82. doi: 10.1016/j.csbj.2022.07.023 35891794 PMC9304428

[58] Nabet B, Roberts JM, Buckley DL, Paulk J, Dastjerdi S, Yang A, et al. The dTAG system for immediate and target-specific protein degradation. Nat Chem Biol. 2018 May;14(5):431–41. doi: 10.1038/s41589-018-0021-8 29581585 PMC6295913

