# Supplementary figures and images for: Resf1 is a compound G4 quadruplex-associated tumor suppressor for triple negative breast cancer
Source: PLoS Genet. 2024 May 9;20(5):e1011236. doi: 10.1371/journal.pgen.1011236 (PMC11081379; doi:10.1371/journal.pgen.1011236)

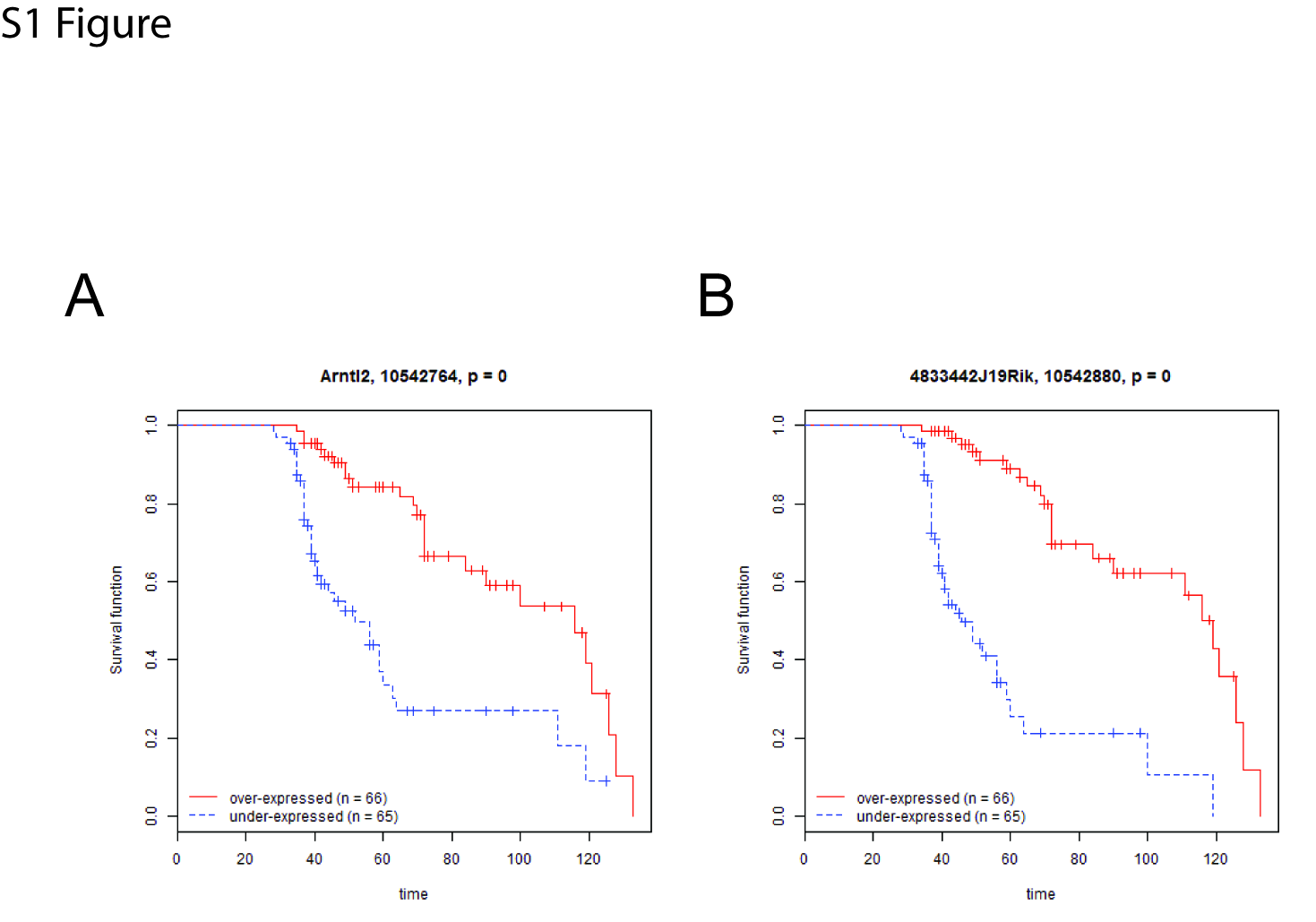

Supplement: S1 Fig — From the N2 backcross cohort in Fig 1C, 131 mice were categorized into over and under-expression of Arntl2 (A), Resf1 (281047O19Rik) (B), and Etfbkmt (4833442J19Rik) (C). Kaplan-Meier curves show worse survival for DMFS when under-expressed. (TIF) [file pgen.1011236.s001.tif]

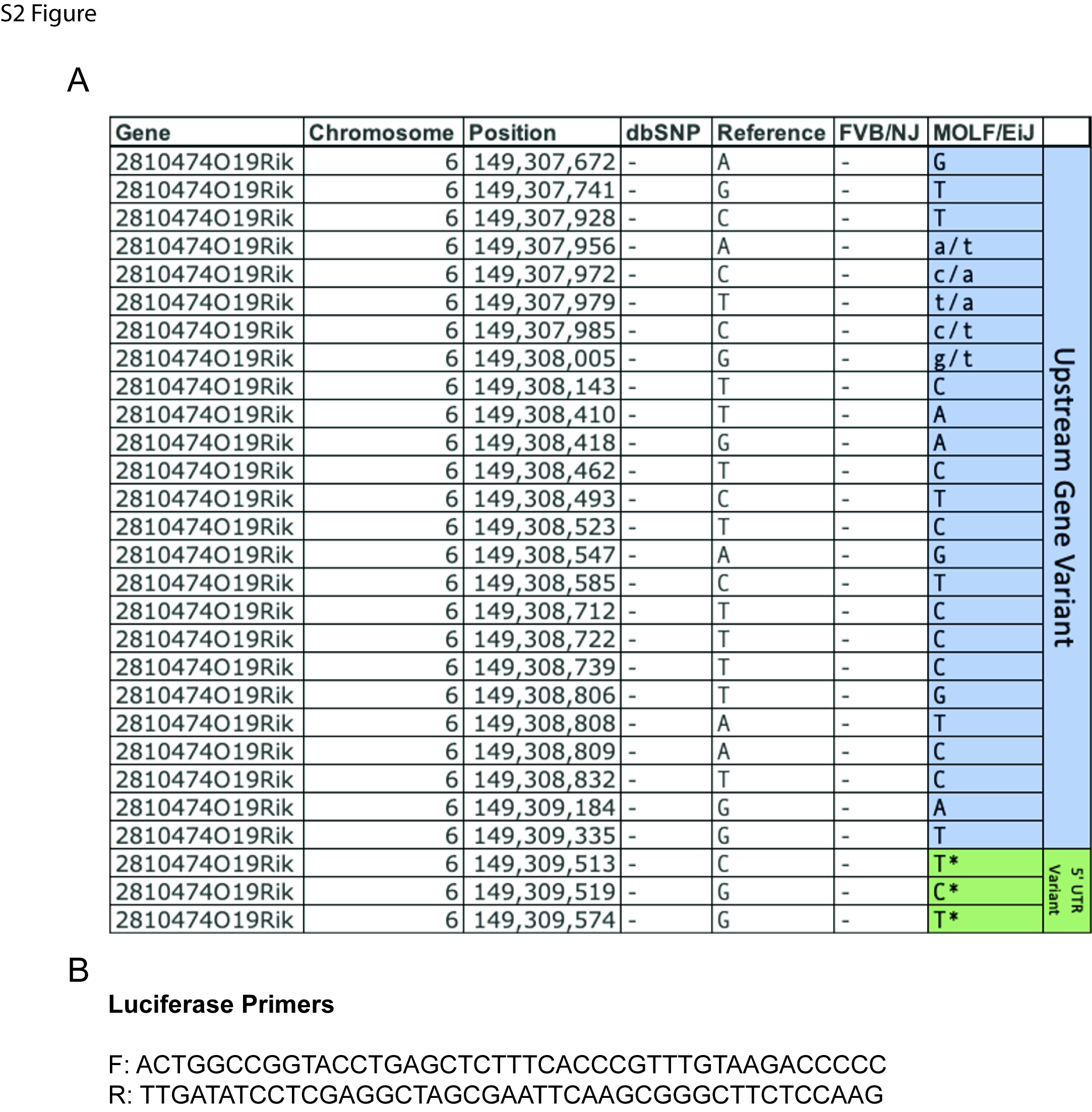

Supplement: S2 Fig — (A) Query of the UCSC BLAT Genome Browser for the 5’ UTR and upstream region of Resf1 displays DHS peaks (green) in the highlighted yellow area. Included are locations of primers used for PCR and cloning of the promoter enhancer region. (B) Primers used for cloning the upstream region of Resf1 into pGL4.23 luciferase reporter plasmid. (TIF) [file pgen.1011236.s002.tif]

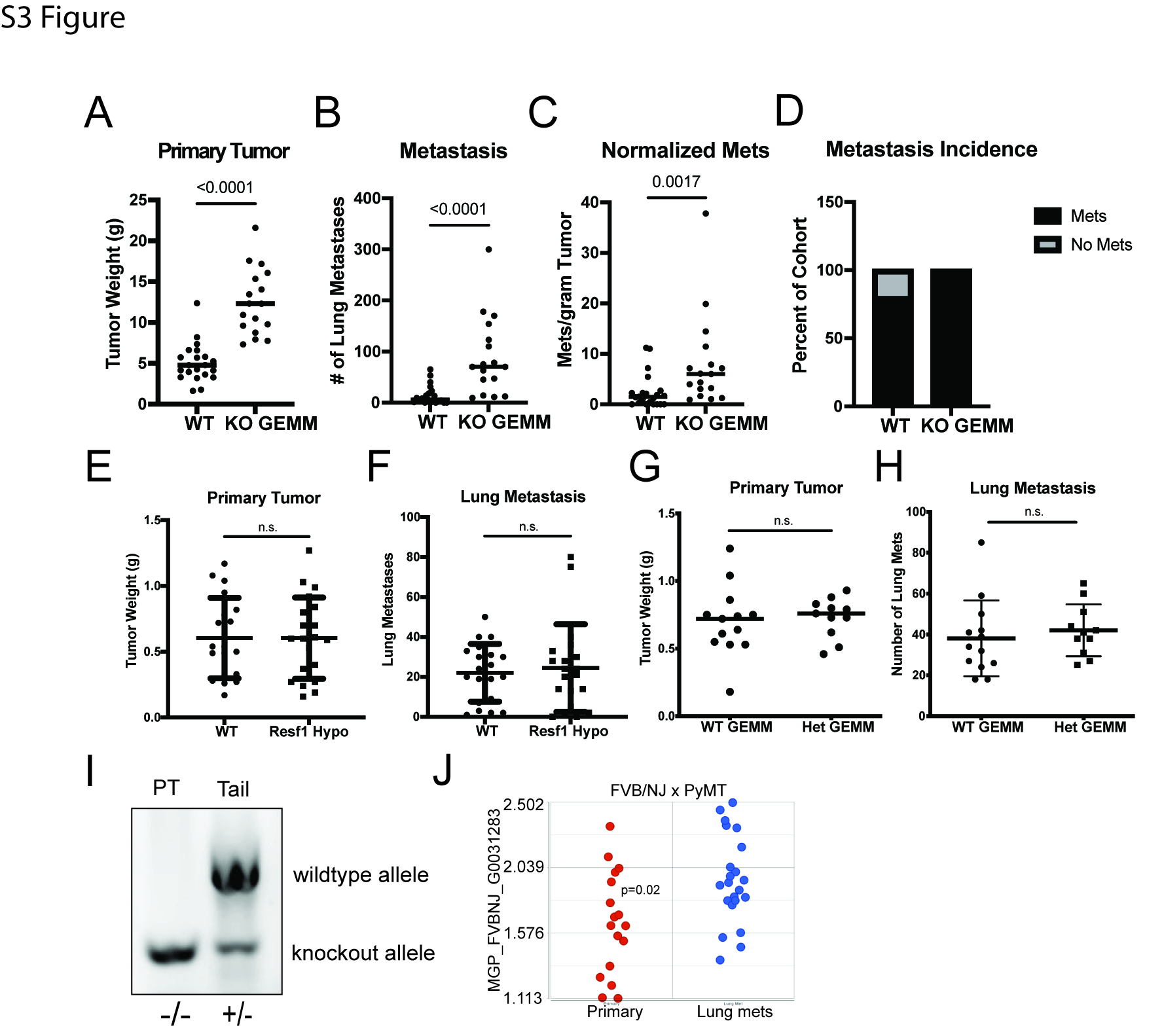

Supplement: S3 Fig — Control and Resf1 CRISPR KO mice were crossed with MMTV-PyMT mice to induce spontaneous mammary tumors and pulmonary metastases, which were allowed to grow for 120 days (A-D). (A) Primary mammary fatpad tumors were collected and weighed for WT (n = 21) and KO (n = 17) mice and resulted in significantly larger tumors in KO mice, with p-value calculated by the Mann-Whitney test. (B) Surface lung metastases were counted, resulting in more metastases in KO mice, p-value calculated by the Mann-Whitney test. (C) Normalization of lung metastases per gram tumor to account for larger tumor size remained significant in KO mice, with p-value calculated by the Mann-Whitney test. (D) Metastatic incidence was higher in KO mice compared to control. Orthotopic injection into the 4th mammary fatpad with syngeneic Mvt1 cells into Resf1 control and hypomorph mice displayed no change in tumor burden or metastasis (E-F). Identical orthotopic injection with syngeneic 6DT1 cells into Resf1 control and KO mice also displayed no change in tumor burden or metastasis (G-H). (I) A representative image of the genotyped tail and primary tumor for Resf1 from WT and KO mice shows a loss of a wildtype allele in primary tumors. (J) Resf1 mRNA expression is higher in lung metastases compared to primary tumors in FVB/NJ x PyMT mice. (TIF) [file pgen.1011236.s003.tif]

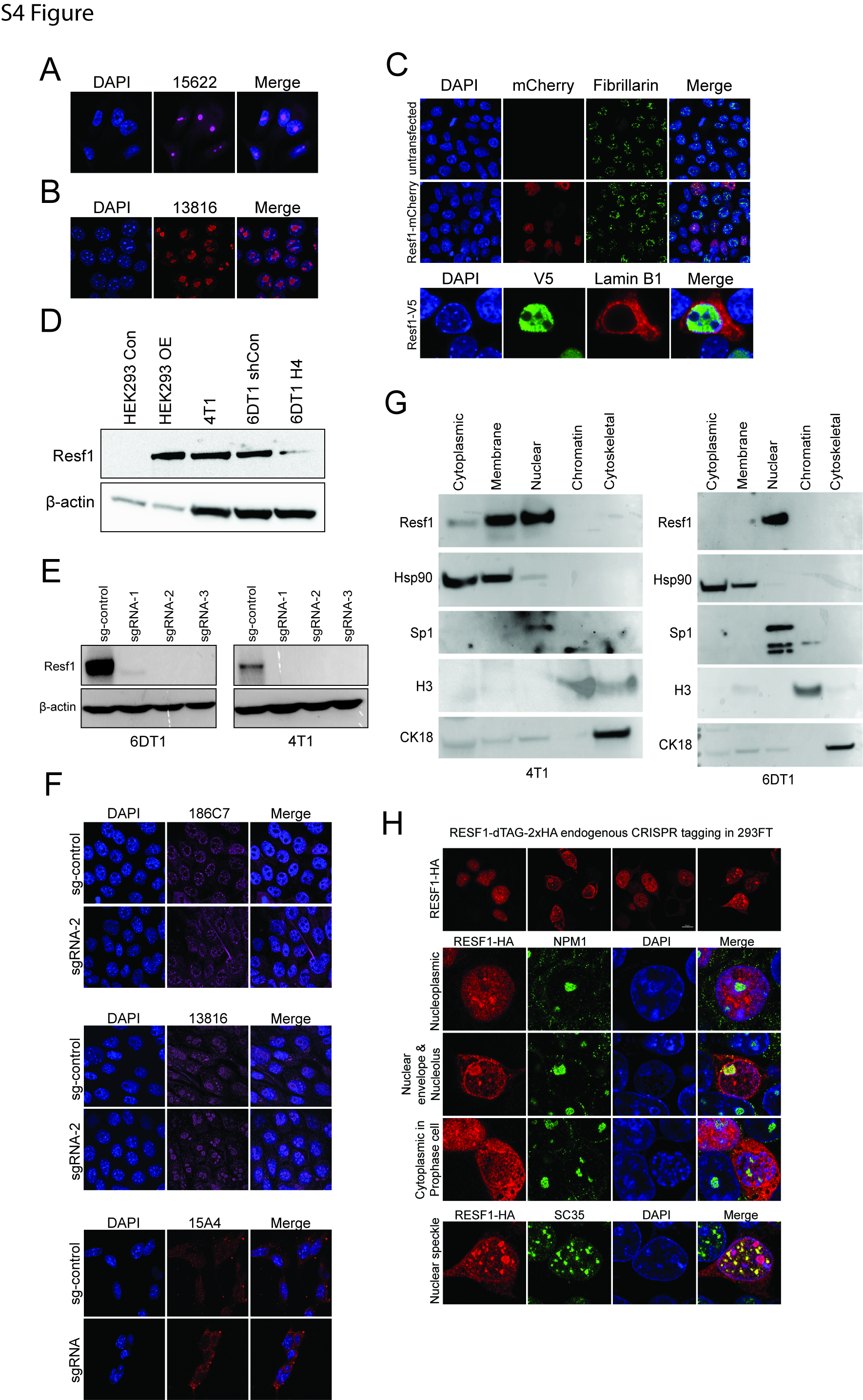

Supplement: S4 Fig — Confocal microscopy images showing nucleolar staining of (A) 15622 antibody, (B) 13816 antibody, and (C) transient transfections of Resf1-mCherry and -V5 in HEK293T cells showing nucleoplasmic staining. (D) Western blot analysis validating custom-generated rabbit antibody raised against mouse Resf1. (E) Western blot analysis showing CRISPR KO of Resf1 in 6DT1 and 4T1 cells. (F) Confocal microscopy images of 4T1 CRISPR KO cells show no differences in 186C7 (4T1), 13816 (4T1), and 15A4 (6DT1) staining between control and sgRNA KO. (G) Subnuclear fractionation western blot analysis of 4T1 and 6DT1 parental cells reveal Resf1 in the nucleoplasm, validated by the controls (Hsp90, Sp1, H3, and CK18) for each subcellular compartment. (H) Confocal microscopy images of RESF1-dTAG-HA knockin in HEK293T cells, co-stained with either NPM1 or SC35. (TIF) [file pgen.1011236.s004.tif]

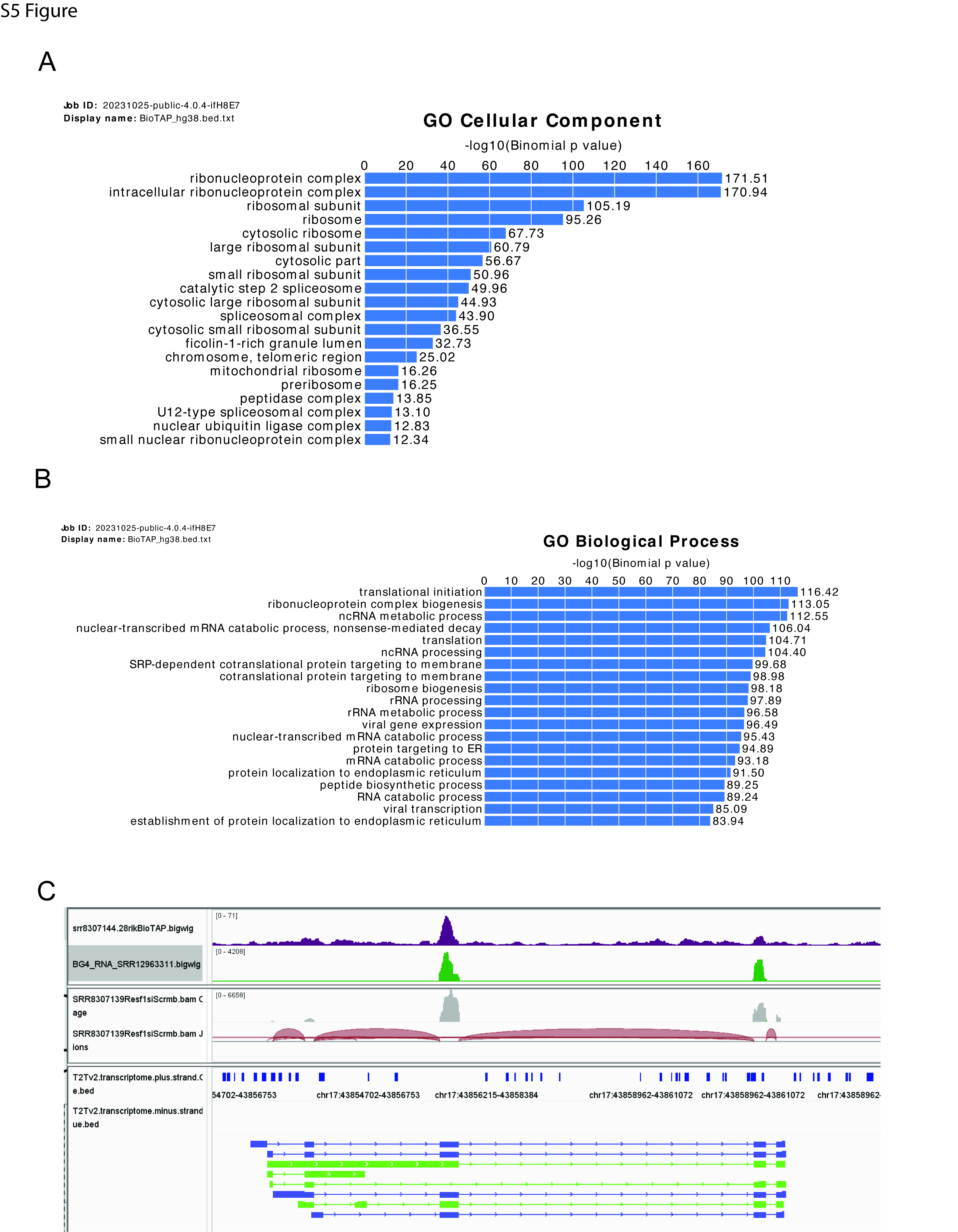

Supplement: S5 Fig — (A) Gene Ontology analysis showing enrichment in cellular components and (B) biological processes involved with translation, noncoding RNA, and ribosomal biogenesis. (C) Mapping of G4 quadruplexes in cytoplasmic RNA and RPL27 mRNA. (TIF) [file pgen.1011236.s005.tif]

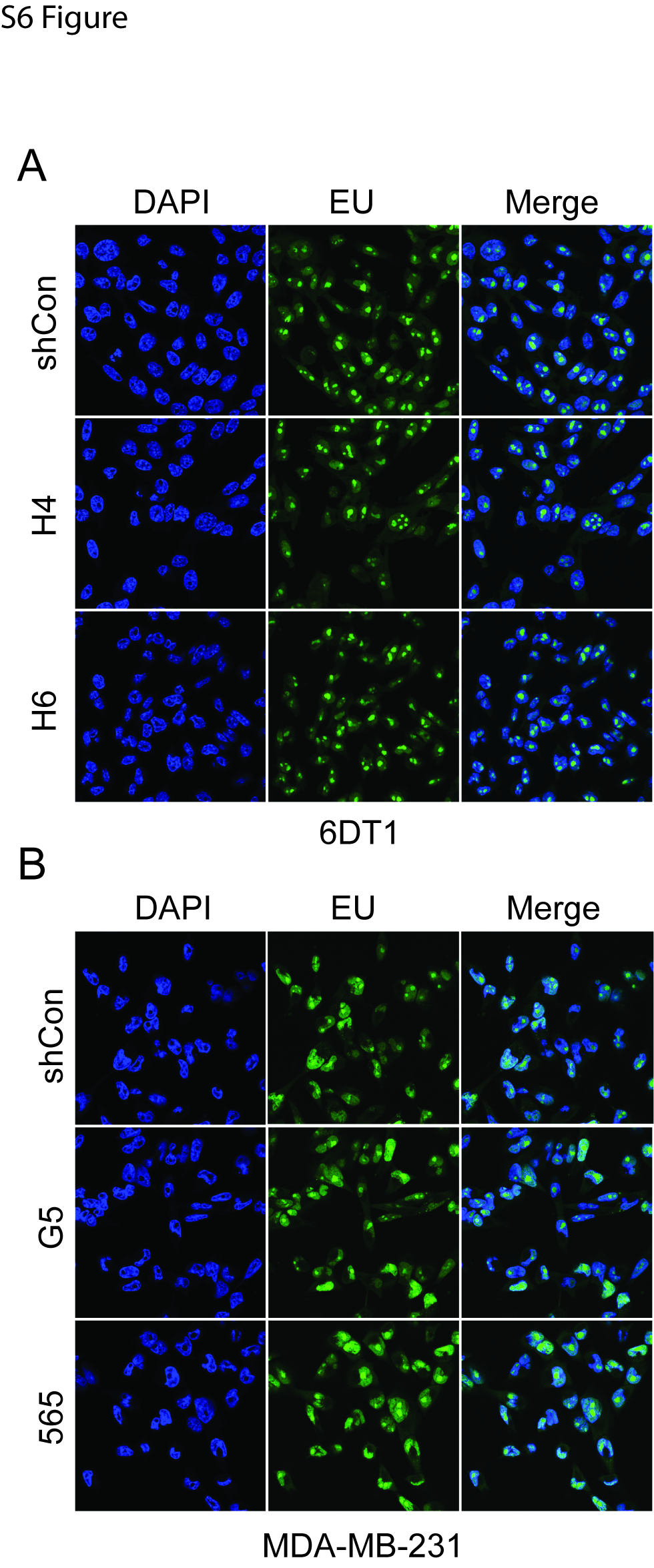

Supplement: S6 Fig — Confocal microscopy images of (A) 6DT1 shRNA KD and (B) MDA-MB-231 shRNA KD cell lines with a 30-minute EU (ethinyl uridine) pulse. (TIF) [file pgen.1011236.s006.tif]

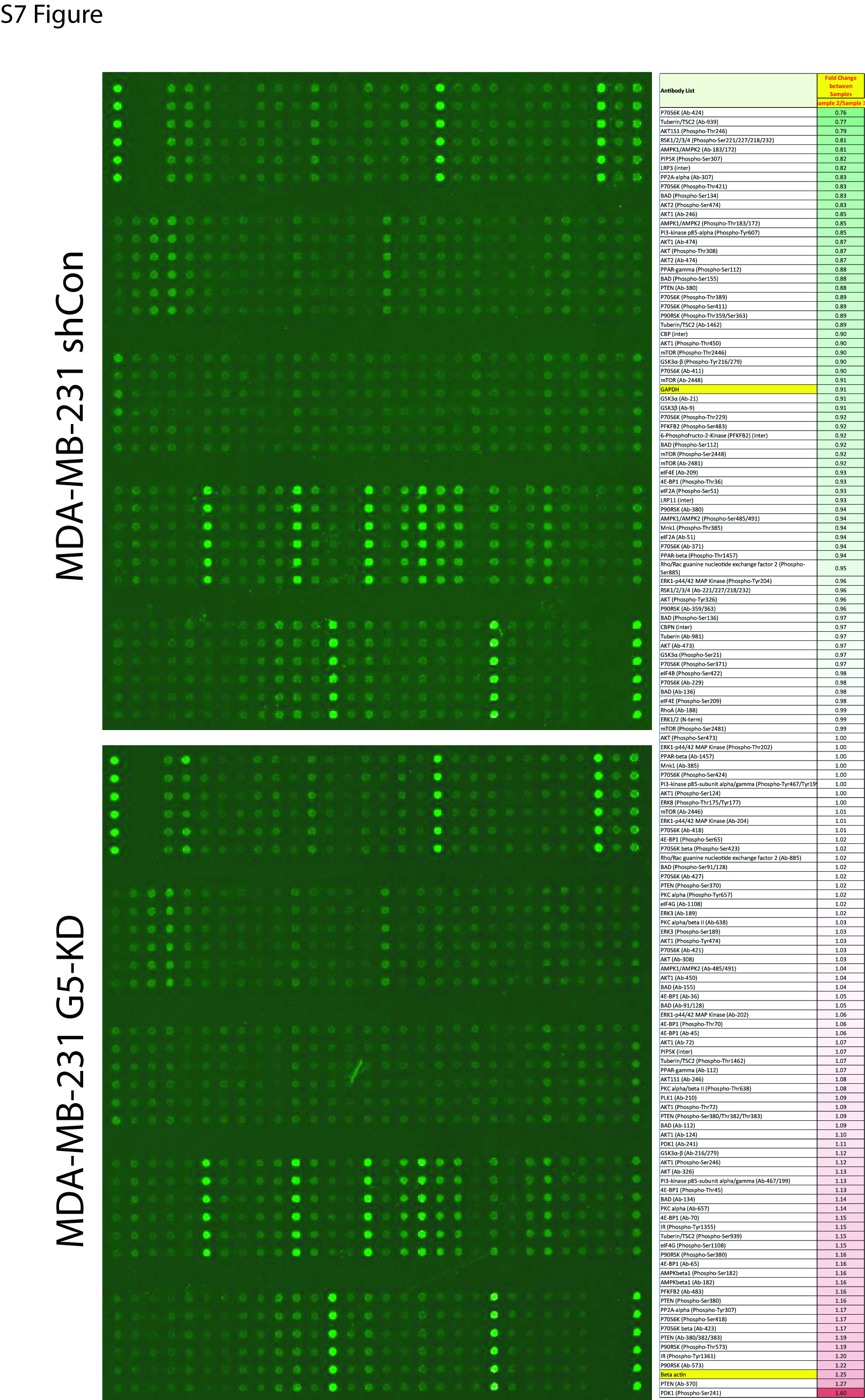

Supplement: S7 Fig — (A) mTOR array shows minimal fold changes in various proteins mTOR pathways upon RESF1 KD in MDA-MB-231 cells. (TIF) [file pgen.1011236.s007.tif]

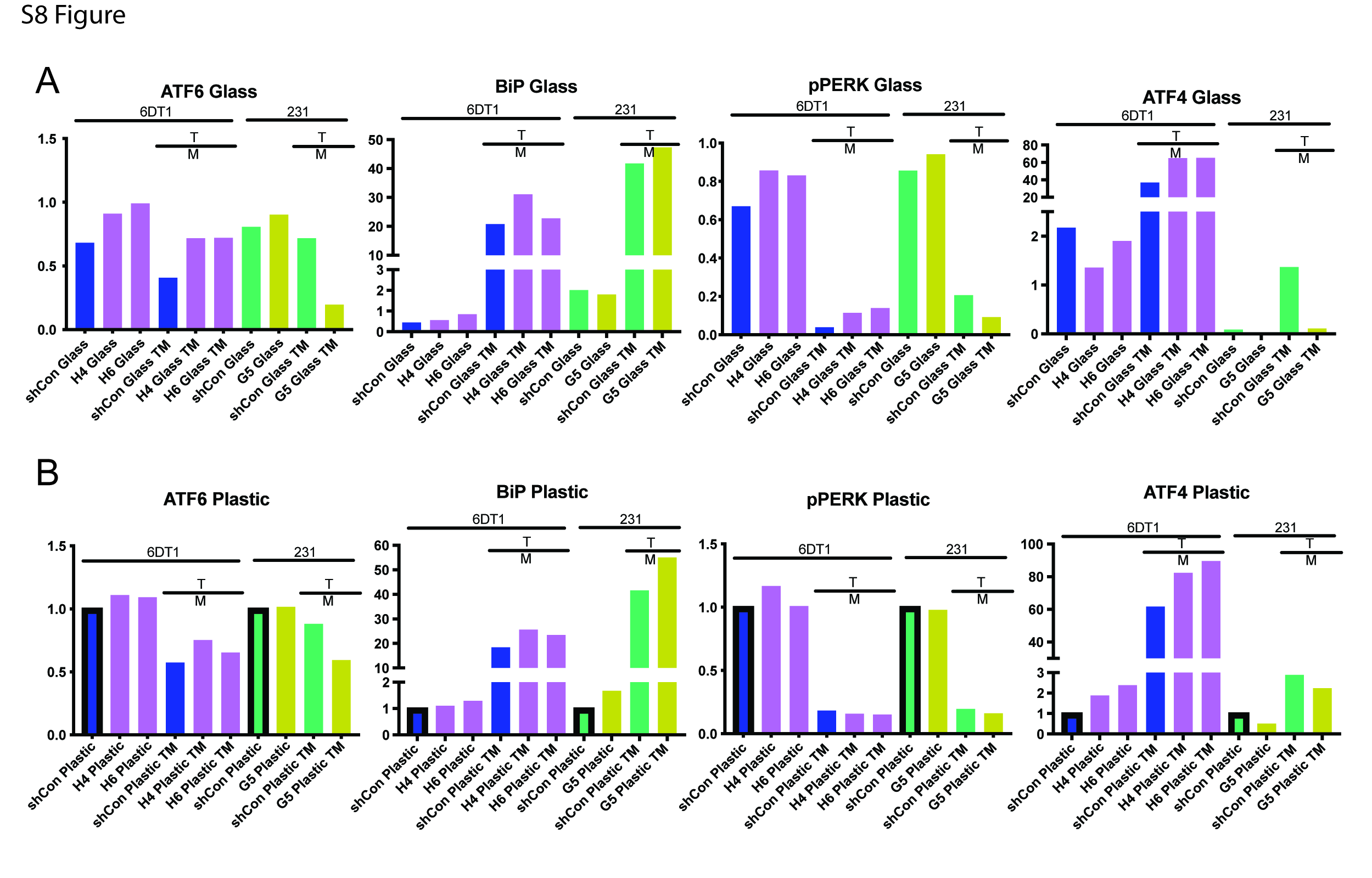

Supplement: S8 Fig — Western blot analysis of several unfolded protein response (UPR) targets in 6DT1 (blue and purple) and MDA-MB-231 (green and yellow), with and without prior tunicamycin (TM) treatment for activation, and glass (A) or plastic (B) culture surface, shows no consistent change in RESF1 KD cells compared to control. (TIF) [file pgen.1011236.s008.tif]

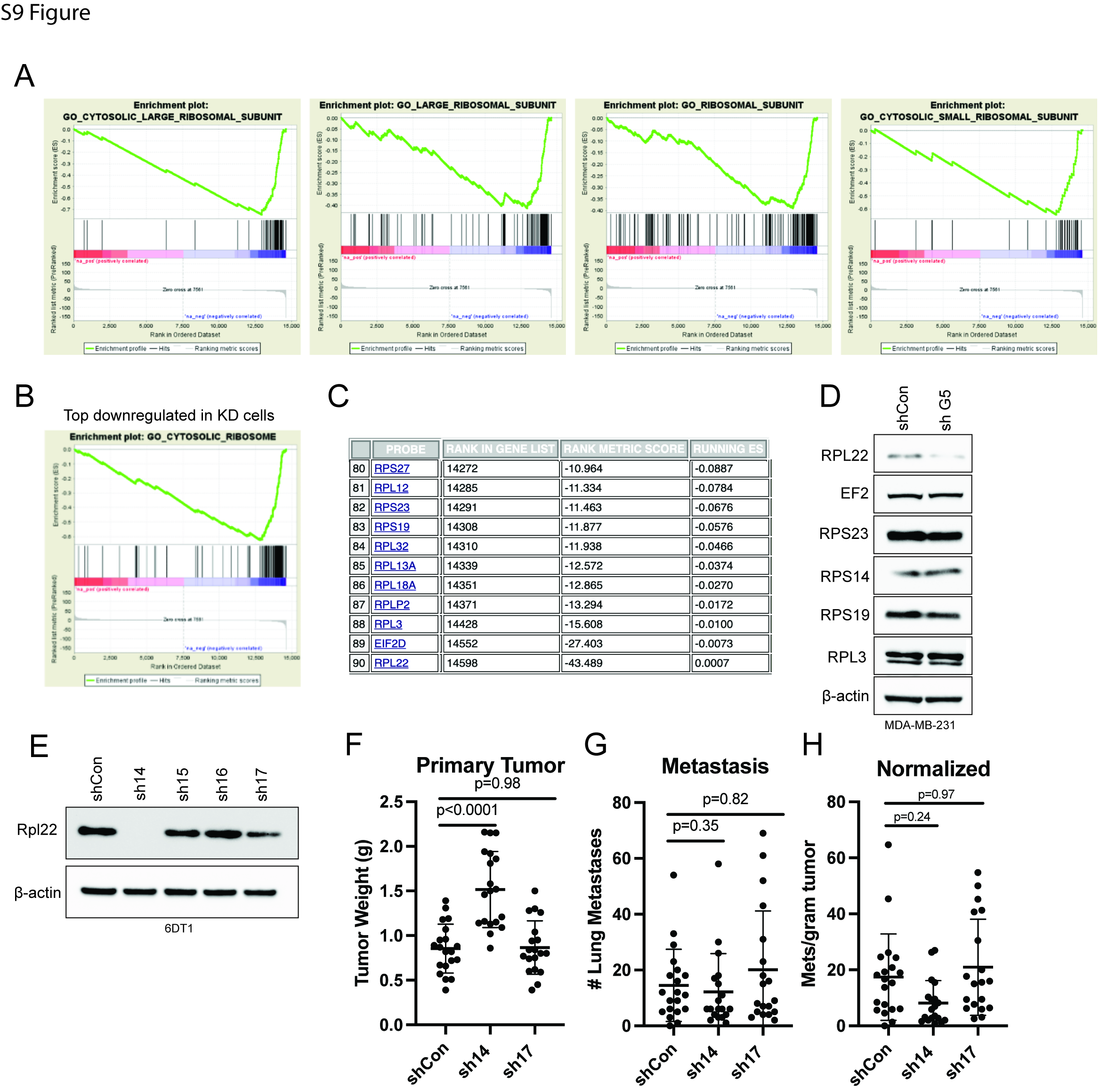

Supplement: S9 Fig — (A) GO Pathway analysis snapshots of various ribosomal subunit pathways that are decreased in 6DT1 Resf1 KD cells. (B) The GO Cytosolic Ribosome pathway had many (C) small and large ribosomal proteins decreased in Resf1 KD cells. (D) Western blot analysis of many of these proteins identified only Rpl22 decreased at the protein level as well. (E) Western blot analysis of 6DT1 shRNA-mediated Rpl22 stable KD cells. (F) Weight of primary tumors from 6DT1 Control (scramble), sh14, and sh17 cells orthotopically injected into the 4th mammary fatpad of syngeneic FVB/NJ mice, n = 20 mice per group. (G) Surface pulmonary metastasis in mice from (F). (H) Pulmonary metastases normalized per gram tumor from (F). P-value was calculated by Mann-Whitney test. (TIF) [file pgen.1011236.s009.tif]

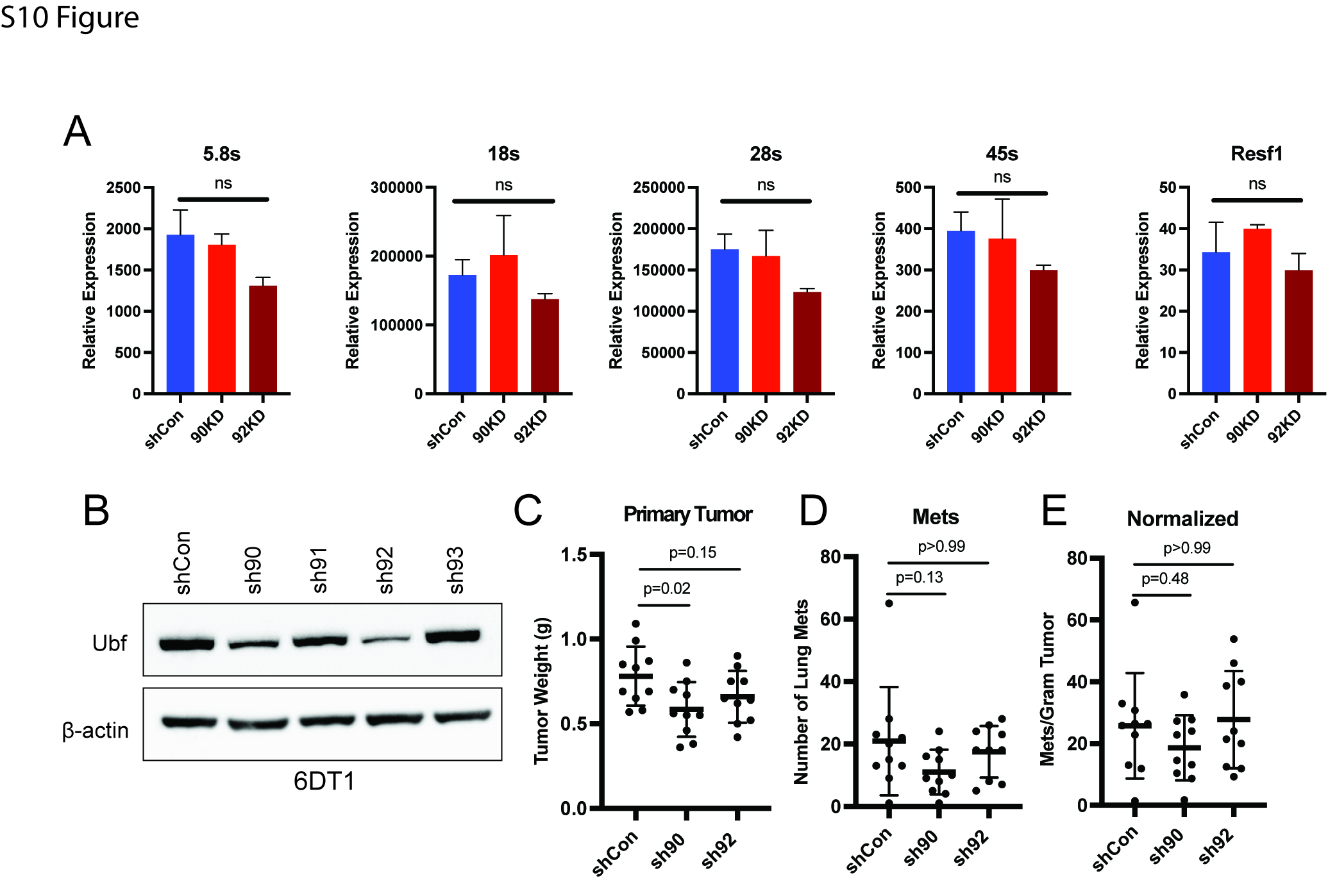

Supplement: S10 Fig — (A) RT-qPCR analysis of 6DT1 Ubf control and KD cells reveals a modest but not significant decrease in rRNA subunits and (B) no change in Resf1 expression levels. (C) Western blot analysis of 6DT1 shRNA-mediated Ubf stable KD cells. (D) Weight of primary tumors from 6DT1 Control (scramble), sh90, and sh92 cells orthotopically injected into the 4th mammary fatpad of syngeneic FVB/NJ mice, n = 10 mice per group. (E) Surface pulmonary metastasis in mice from (D). (F) Pulmonary metastases normalized per gram tumor from (D). P-value was calculated by Mann-Whitney test. (TIF) [file pgen.1011236.s010.tif]

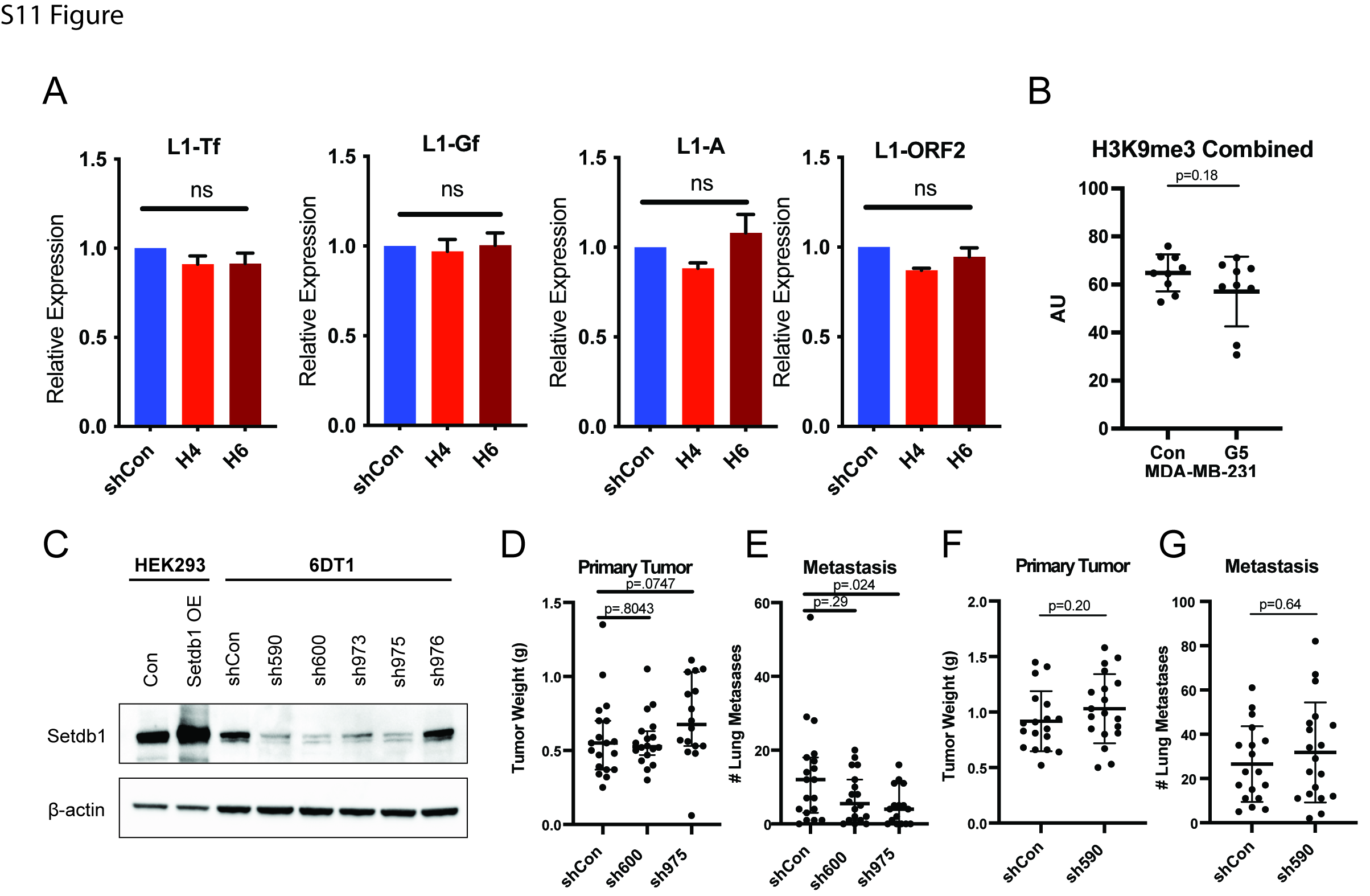

Supplement: S11 Fig — (A) RT-qPCR analysis of three different families of LINE (L1) elements display relatively no change upon Resf1 KD in 6DT1 cells. (B) A colorimetric H3K9me3 capture based assay also displayed no significant change in global tr-methyl histone H3K9 between control and RESF1 KD MDA-MB-231 cells, p-value based on Mann-Whitney test. (C) Setdb1 protein expression in 6DT1 cells shown by western blot, with a transiently transfected overexpression control in 293FT cells. (D) Weight of primary tumors from 6DT1 Control (scramble), 600KD, and 975KD cells orthotopically injected into the 4th mammary fatpad of syngeneic FVB/NJ mice, n = 20 mice per group, combined data from 2 experiments. (E) Surface pulmonary metastases counted from (D). Weight of primary tumors from 6DT1 Control (scramble), 590KD cells orthotopically injected into the 4th mammary fatpad of syngeneic FVB/NJ mice, n = 20 mice per group, combined data from 2 experiments. (G) Surface pulmonary metastases counted from (F). All p-values calculated by Mann-Whitney test. (TIF) [file pgen.1011236.s011.tif]

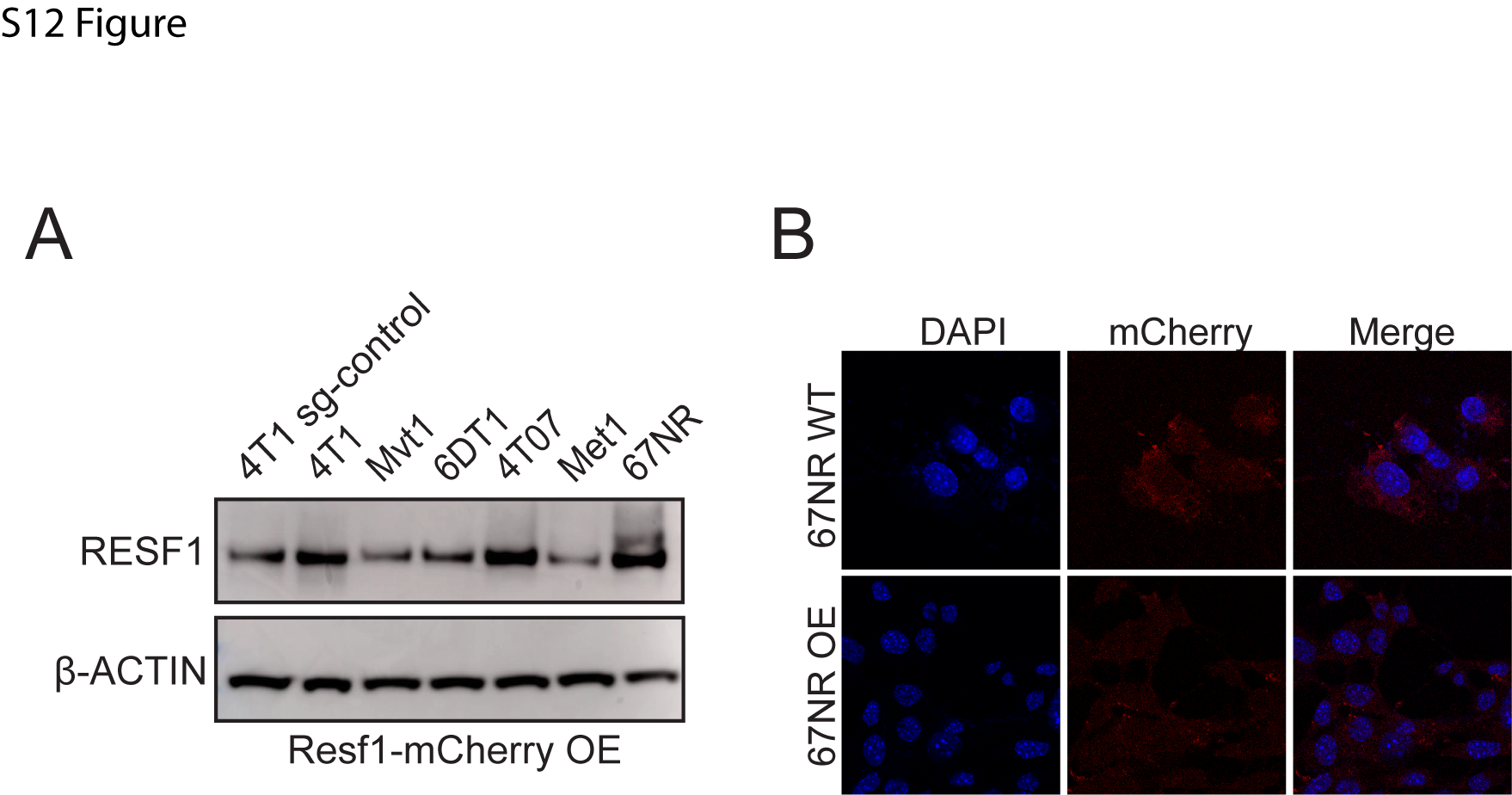

Supplement: S12 Fig — (A) Western blot analysis reveals stable expression of RESF1 in 67NR cell line, as shown with Resf1-mCherry construct. (B) Immunofluorescence analysis showing no specific mCherry signal in 67NR WT and overexpressed cell lines. (TIF) [file pgen.1011236.s012.tif]
