# Supplementary material for: Resf1 is a compound G4 quadruplex-associated tumor suppressor for triple negative breast cancer
Source: PLoS Genet. 2024 May 9;20(5):e1011236. doi: 10.1371/journal.pgen.1011236 (PMC11081379; doi:10.1371/journal.pgen.1011236)
Supplement: S1 Table — (PDF) [file pgen.1011236.s013.pdf]

qPCR oligos	Sequence
Resf1 mouse	Forward: 5’-CATGTGCAGAATGCTCAGCC-3’<br>Reverse: 5’-TTAGGGTTCTGCCACTGCTG-3'
RESF1 human	Forward: 5’-GAAGAGCCCATCACAGAAGTAG-3'<br>Reverse: 5’-ATGCACAGCAGTGGATATCAT-3'
PPIB	Forward: 5’-AGCTACAGGAGAGAAAGGATTTGGC-3’<br>Reverse: 5’-AAGCGCTCACCATAGATGCTC-3'
Epcam	Forward: 5'-AATGCCAGTGTACTTCCTATG-3’<br>Reverse: 5'-CTTTATCCTCCTCCCAGACT-3'
Vimentin	Forward: 5'-CTCGTCACCTTCGTGAATAC-3’<br>Reverse: 5'-TCTCAGGTTCAGGGAAGAA-3'
18S rRNA	Forward: 5'-CGGCTACCACATCCAAGGAA-3’<br>Reverse: 5'-GCTGGAATTACCGCGGCT-3'
28S rRNA	Forward: 5'-CGACGACCCATTCGAACGTCT-3’<br>Reverse: 5'-CTCTCCGGAATCGAACCCTGA-3'
5.8S rRNA	Forward: 5'-ACTCTTAGCGGTGGATCACTC-3’<br>Reverse: 5'-AAGCGACGCTCAGACAGG-3’
45S pre-rRNA	Forward: 5'-CGTGTAAGACATTCCTATCTCG-3’<br>Reverse: 5'-GCCCGCTGGCAAGAACGAGAAG-3'
L1-Tf	Forward: 5'-CAGCGGTCGCCATCTTG-3’<br>Reverse: 5'-CACCCTCTCACCTGTTCAGACTAA-3'
L1-Gf	Forward: 5'-CTCCTTGGCTCCGGGACT-3’<br>Reverse: 5'-CAGGAAGGTGGCCGGTTGT-3'
L1-A	Forward: 5'-GGATTCCACACGTGATCCTAA-3’<br>Reverse: 5'-TCCTCTATGAGCAGACCTGGA-3'
L1-ORF2	Forward: 5'-GGAGGGACATTTCATTCTCATCA-3’<br>Reverse: 5'-GCTGCTCTTGTATTTGGAGCATAGA-3'
**RNA FISH oligos**	**Sequence**
5.8S rRNA	/5Cy3/CAUCGACGCACGCACGAGCCGAGUGAUCCAC
18S rRNA	/5Cy3/GAGGUUUCCCGUGUUGAGUCAAAUUAAGCCGCA
28S rRNA	/5Cy3/ACGGGCUGGGCCUCGAUCAGAAGGACUUGG
**G4 oligos**	**Sequence**
RPL27 G4.1	5'-GCCCATGGCAGCTGTCACTTTGCGGGGGTAGCGGTCAATT-3'
RPL27 G4.2	5'-GAGCATGGCTGTAGGGGCGATCTGAGGTGCCAT-3'
